# The alpha rhythm: from physiology to behaviour

**DOI:** 10.1152/physrev.00001.2025

**Published:** 2026-02-03

**Authors:** Ole Jensen, Mathilde Bonnefond

**Affiliations:** 1 Oxford Centre for Human Brain Activity, Wellcome Centre for Integrative Neuroimaging, Department of Psychiatry, University of Oxford, Oxford, OX3 7JX, UK; 2 Department of Experimental Psychology, University of Oxford, Oxford, OX2 6GG, UK; 3 Université Claude Bernard Lyon 1, CNRS, INSERM, Centre de Recherche en Neurosciences de Lyon CRNL U1028 UMR5292, COPHY, F-69500, Bron, France

## Abstract

The alpha rhythm, first identified by Hans Berger 100 years ago, is the dominant non-invasive electrophysiological signature of the healthy human brain in the awake state. For decades, it was believed that the alpha rhythm reflected rest or idling; however, this perspective changed in the 2000s when researchers found that alpha oscillations increase with cognitive demands. This discovery led to a paradigm shift, demonstrating that alpha oscillations reflect the functional inhibition of brain regions that are not needed for a specific task, thereby directing information to task-specific areas. We have reviewed the physiological mechanisms involved in generating alpha oscillations, which has informed computational models explaining how these oscillations emerge within physiologically realistic networks. At the behavioural level, alpha oscillations are strongly modulated across nearly all cognitive paradigms tested in humans, reflecting the allocation of computational resources within the active brain network. Research in individuals with attention-related issues has highlighted their impaired ability to modulate alpha oscillations, which is associated with performance deficits. Therefore, further exploration of alpha oscillations has the potential to uncover causal mechanisms underlying attention problems, such as those related to ADHD and ageing. Lastly, advancements in technology are opening new avenues for characterising alpha oscillations in ecologically valid settings and across the lifespan. This progress sets the stage for exploring the role of alpha oscillations in cognitive development and their functioning in natural environments.

## Graphical abstract

**Graphical abstract:**
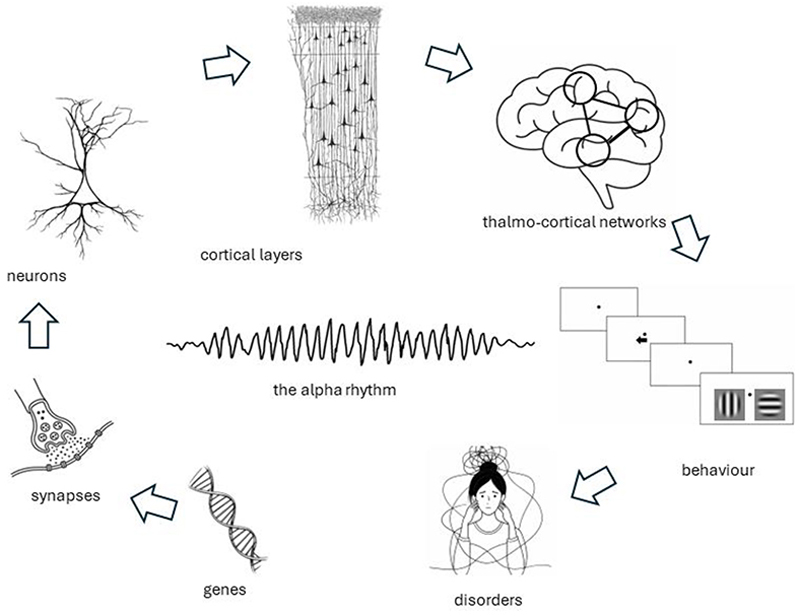

## 1 Introduction

The alpha rhythm is one of the most prominent electrophysiological signals that can be measured non-invasively from the awake healthy human brain. Discovered 100 years ago by Hans Berger, the alpha rhythm has been the focus of extensive research aimed at uncovering its functional role in cognition, as well as its relevance in neurological and psychiatric disorders. In this review, we will first provide a historical overview of the research on alpha oscillations and discuss some of the debates surrounding their origin and functional role. For a long time, alpha oscillations were thought to reflect a state of rest or idling. However, in recent decades, this perspective has shifted to recognize that alpha oscillations actively contribute to the functioning brain by inhibiting regions that are not required for specific tasks. Aligned with this insight, we will explore the insights gained on the physiological mechanisms that generate alpha rhythms, along with the computational models developed to explain the emergence of these oscillatory dynamics. We will then discuss the cognitive paradigms used to investigate the functional role of alpha oscillations. Because most tasks applied in the cognitive paradigms modulate alpha oscillations, our focus will be on the core mechanisms and networks that generate and regulate these rhythms at the systems level. Additionally, this review will include clinical considerations regarding the role of alpha oscillations in individuals experiencing attention and memory problems. Lastly, we will highlight emerging themes and suggest future research directions where the study of alpha oscillations appears particularly promising. We argue that the connection between alpha oscillations and saccadic control has been underappreciated as well as the genetic basis of the rhythm generation. Furthermore, research on brain oscillations within developmental contexts could provide valuable insights, given that many disorders and cognitive problems have their onset in early childhood.

## 2 A century of research on the alpha rhythm: from idling to inhibition

### 2.1 Hans Berger’s seminal contribution

The alpha rhythm was discovered by Hans Berger in 1924, and he published the first papers on the topic in 1929 (1). This followed years of dedicated experimentation in which he developed and applied the technique of electroencephalography (EEG). What drove Berger to discover the alpha rhythm - was it a chance discovery or was his quest anchored in a broader vision?

Hans Berger was educated as a psychiatrist and spent most of his career at Jena University Hospital where he eventually was appointed Professor of Psychiatry and Director of the University Clinic (2). While his research was not driven by a desire to explain the physiological basis of psychiatric disorders *per se*, his work at a hospital gave him the chance to embark on research on human brain physiology using electrophysiological approaches. Hans Berger was inspired by the idea that mental processes would be associated with energy transfer within the brain as previously argued by (3) and (4). These ideas were derived from developments in physics where the notions of energy transfer and preservation formed a theoretical foundation. In particular, Lehmann (1892) had conjectured that chemical energy in the brain was converted into heat, electricity and psychic energy. In line with this thinking, Berger hoped to identify the physiological signals reflecting psychic energy. He initially investigated the blood flow in the brain in relation to various attention-demanding tasks. This was done in patients that had skull defects or undergone craniotomies allowing for measuring the pulsating brain through the skull. Measurements were done by applying a plethysmograph over where the skull was removed while the patient performed various tasks or drug challenges such as cocaine (5). Later he embarked on measuring small changes in brain temperature in response to different manipulations (6). This was done by placing thermometers in the brain requiring a cerebral puncture done under local anaesthesia. These studies were inspired by ideas on brain activity producing temperature changes due to energy transfer (7). The studies that Berger conducted on blood flow and temperature changes turned out not to be particularly informative as they did not produce stable results. Nevertheless, Berger was also interested in electrical brain recordings. He was among the first to perform recordings from the cortical surface of dogs using a capillary electrometer designed to measure the electrocardiogram. Later he used a more sensitive instrument, namely an Edelmann string galvanometer and conducted further experiments in dogs measuring response to various sensory stimuli (reviewed in (8). These studies were of limited success but eventually, Berger applied the technique to measure the EEG from the human scalp. This was first done in patients with parts of the skull removed and later from colleagues and family members. After several years of refining the techniques and collecting data, he was eventually able to obtain reliable results and referred to the ongoing electrical scalp data as the Elektroenzephalogramm (in English the *electroencephalogram*; *EEG*). Using this approach, he characterised awake and sleep stages and abnormal EEG activity associated with epilepsy. In the awake studies, he identified ∼10 Hz oscillations that he labelled the alpha rhythm as well as ∼20 Hz oscillations that he labelled the beta rhythm. He then conducted a series of studies to describe how the alpha rhythm is modulated in response to sensory stimuli and attention-demanding tasks. Typically, the alpha oscillations decreased with visual stimulation and attention. Importantly, he observed an increase in the magnitude of the brain rhythms over trepanations. This observation strengthened the case that the measured signals were generated in the brain rather than produced by e.g. muscle artefacts. These findings were revolutionary, and Hans Berger is unequivocally considered the discoverer of the alpha rhythm, however, he only received limited recognition from peers in his time. He did however live to see his findings reproduced and supported by (9). Hans Berger took his own life in 1941 after struggling with congestive heart failure, a painful skin disease and depression. One might ask why Berger did not receive more recognition for his achievements at an earlier stage. It should be mentioned that Hans Berger was known as a stern and highly diligent clinician, and he carried out most of his scientific work independently. He did not establish collaborations or build a research group paving the path for the younger generation to continue his work (10). At the same time, other contemporary researchers interested in the physiology of the brain were focussing on assigning functions to specific brain areas. This perspective did not resonate well with Berger’s thinking as he believed the alpha rhythm reflected a more general state generated across the brain. Indeed, Berger was criticised for not attempting to localise the regions producing the reported brain oscillations (11). Also, the papers of Hans Berger were written in a complicated writing style and therefore less accessible to the broader international research community. While there has been much speculation on why Berger did not receive more recognition during his lifetime (12), one should probably not underestimate the fact that Berger had a demanding clinical position as a psychiatrist and eventually became the Director of the Jena University Clinic and was later elected Rector of Jena University. These positions would have limited the time and energy he could dedicate to research. Furthermore, the political situation in Germany was evidently tumultuous and the country was becoming increasingly isolated during the decades when Berger developed his research. These factors cannot have helped the dissemination of his work. Nevertheless, beyond the discovery of alpha oscillations, the invention of EEG became a standard tool for diagnosing epileptic activity in patients (Golla *et al*., 1937; Walter *et al*., 1951) and for quantifying sleep stages (13).

In conclusion, Berger’s research resulted in the discovery of the alpha rhythm, and his endeavours were anchored in a framework exploring energy transfer associated with mental work. While his initial works on blood flow and temperature changes were inconclusive, one could argue he was on the right track. Indeed, the positron emission tomography (PET) studies starting the revolution of modern-day brain imaging were centred around measuring metabolism and energy consumption in response to sensory stimulation and attentionally demanding tasks (14). The field of functional magnetic resonance imaging (fMRI) is based on measuring blood flow changes associated with the engagement of specific brain regions in response to cognitive tasks (15). As we will review later, there are strong relations between modulations in the blood-oxygen-level-dependent (BOLD) signal as measured by fMRI and the alpha rhythm as measured by EEG.

### 2.2 Adrian and Matthews substantiating the findings on the alpha rhythm

**Figure 1:**
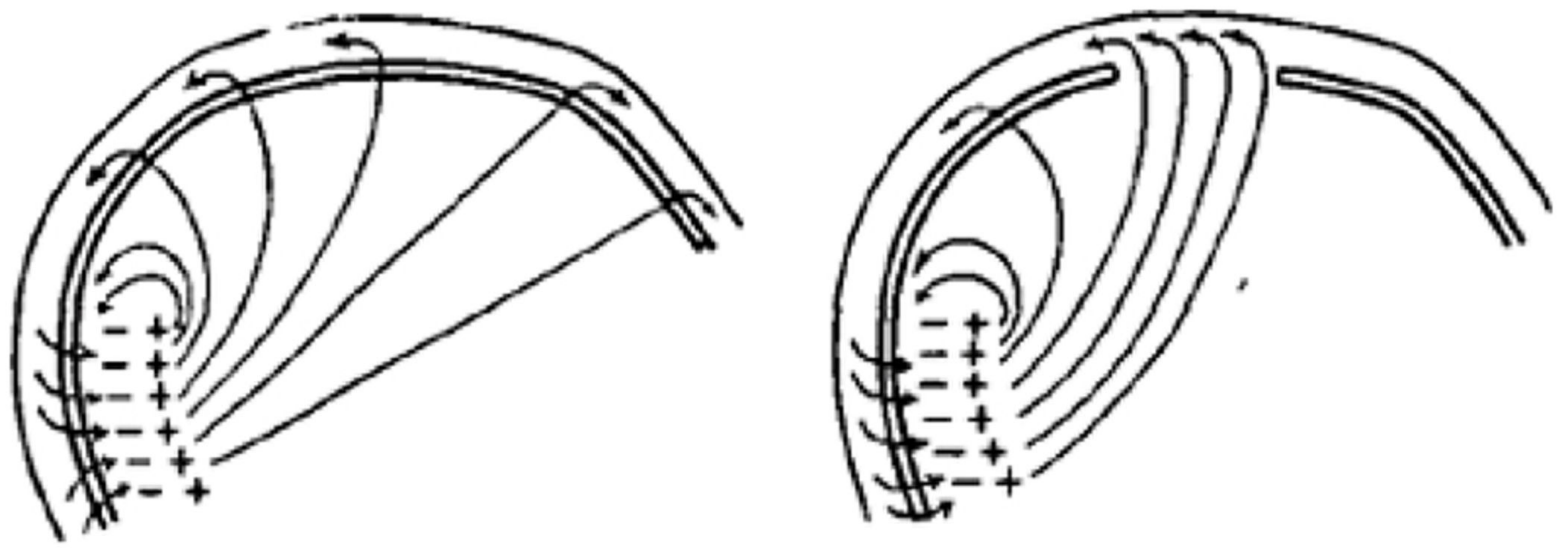
The model explaining why the alpha rhythm is spatially distributed on the human scalp even if the sources are in the occipital cortex (left). The model also accounts for why the alpha rhythm appears stronger over trepanations supporting its neuronal origin (right). This framework has stood the test of time and has been formalised in *forward models* used in source analysis. Reproduced from (9); used with permission.

In 1934 the famous physiologist Lord Adrian and his collaborator Matthews conducted a set of experiments on the alpha rhythm using EEG (9). They were initially sceptical about Hans Berger’s findings and set out to address the concerns that the alpha rhythm might be generated by various artefacts rather than in the brain. However, their investigations provided strong support for most of Berger’s claims, and they honoured the initial work by referring to the 10 Hz waves as the *Berger rhythm*. By conducting a larger set of studies, they confirmed that the alpha waves became strong when participants were at rest and did not receive any informative visual input. They demonstrated that alpha oscillations would emerge during uniform visual stimulation, however, if a visual stimulus was presented in the fovea, the rhythm would decrease. They concluded that it was not the visual input *per se* but rather the attention to the visual input that caused the blocking of the alpha rhythm. However, their data did not favour Berger’s claim that the generation of the alpha rhythm was widespread across the brain. Rather, they argued that the alpha rhythm was produced in the extrastriate occipital lobe. This conclusion was based on the posterior distribution of the rhythm and its strong modulation by visual input. As the alpha oscillations could persist in the presence of visual input (as long as it was non-patterned) they excluded a source in the striate cortex. A later study based on recording the EEG from electrodes placed at different scalp locations confirmed the occipital alpha source (16). Adrian and Matthews also presented a case where the patient had parts of the scalp removed before undergoing surgery for a brain tumour (9). This allowed for direct recordings from the brain surface and strong alpha oscillations were observed. They also presented a physiological model for how the current distribution in the brain volume and on the scalp could be generated from potential differences generated by neurons in the posterior part of the brain (Fig. 1). This model could explain why volume conduction will result in the alpha rhythm being widespread across the scalp and also why the signal is stronger over trepanations. This framework remains valid and has been formalised in so-called forward models used in modern-day source analysis (17).

In conclusion, the findings by Berger were confirmed by Adrian and Matthews. While Berger argued that the alpha rhythm was generated throughout the cortex, Adrian and Matthews argued for an extrastriate source in the occipital cortex. As we will later demonstrate, the alpha rhythm does have strong generators in parieto-occipital areas albeit it is now clear that it can also be generated by other parts in the neocortex including parietal, temporal and prefrontal regions. The conclusions by Adrian and Matthews also formed the basis for the notion that the alpha rhythm was produced spontaneously when a network was left unperturbed thus resulting in the thinking that alpha waves reflect a state of rest or idling. It would take more than 60 years for this view to be challenged by the idea that alpha oscillations in fact reflect active inhibition of regions not engaged in a specific task.

### 2.3 Ocular muscle tremors and the alpha rhythm

While the neuronal origins of human alpha oscillations have been questioned by various researchers across time it has stood the test of time. For instance, Lippold proposed that the alpha rhythm might be explained by ocular artefacts generated by tremors in eye muscles (18,19). This suggestion was based on observations in which the physiological manipulations of the eyes were linked to alpha oscillations. For instance, cooling or warming the orbits around the eye would modulate the frequency of the alpha oscillations (20). The displacement of the eyeball by a prodding device resulted in alpha oscillations being elicited in the EEG (21). Lippold’s objections created a strong debate and were later refuted for instance by intracranial recordings revealing neuronally produced alpha oscillations. Nevertheless, the concerns underscored an underappreciated link between alpha oscillations and the eye movement system, which we will discuss below.

### 2.4 Intracranial findings on the alpha rhythm in animals and humans

One of the puzzling findings on the alpha rhythm was that while it appeared highly robust in human scalp recordings, there were mixed results when performing intracranial recordings in both humans and non-human primates. Over the years there have been many reports on alpha oscillation recordings in various species. The first example was recordings from the optic ganglion of the water beetle demonstrating an increase in 10 Hz oscillations when the insect was in dark compared to light environments (9). Later strong alpha oscillations were recorded from the codfish (22). These oscillations increased during darkness and were coupled to the thalamus. There are also reports on alpha oscillations in goldfish, toads and chicken (23–25). While one can question whether these phenomena are equivalent to what is observed in humans, it does open the exciting possibility that the alpha rhythm reflects fundamental physiological properties that are preserved across species and might play a general functional role.

Alpha oscillations have been observed in several mammalian species, including cats, dogs, and ferrets (26–31). Research in dogs was particularly influential in shaping the debate over whether alpha oscillations were primarily generated by the thalamus, as suggested by studies of barbiturate-induced spindle oscillations (32). However, recordings in dogs provided strong evidence for a neocortical origin of the alpha rhythm, distinct from barbiturate-induced spindles (33). Notably, until the early 2000s, there was relatively little interest in studying alpha oscillations in non-human primates. This could be explained by the focus on investigating neuronal spiking rather than local field potentials (LFPs) in non-human primates. Nevertheless, using laminar electrodes, alpha oscillations were reported in V2, V4 and IT of macaque monkeys (34). Interestingly the alpha oscillations in V2 and V4 correlated negatively with performance whereas the opposite was the case in IT. A study relating neuronal spiking to oscillations in the LFPs in V1, V2 and V4 of macaque monkeys found that the spiking was phase-locked to alpha oscillations. Importantly, this phenomenon occurred in deep but not superficial layers of the neocortex (35). This suggests that alpha oscillations are generated in deeper cortical layers and confirmed by applying current source density analysis to laminar recordings in (36,37) (but see (38)). The alpha generators in deep layers would explain why the alpha rhythm is relatively modest in brain surface recordings in animals and humans. The deep-layer generators would be associated with currents in the long dendrites of layer 5 pyramidal cells which would sum up to fields detectable by magnetoencephalography (MEG) and result in additive return currents detectable with EEG. It should also be mentioned that most intracranial recordings in humans are done with the primary purpose of guiding surgical resections in patients with epilepsy or brain tumours. While it is only on rare occasions that electrodes are placed in occipital lobes, there is often good coverage around the central sulcus. Indeed, there are convincing reports on alpha oscillations - the so-called mu-rhythm - being produced in the precentral sulcus (39–41). This rhythm behaves like the posterior alpha oscillations as they are blocked with somatosensory or motor engagement.

In sum, there are now numerous reports on the alpha rhythm being detected intracranially obtained from both animal and human recordings. The initial scarce reports on intracranial human alpha oscillations when recording from the brain surface might be explained by the alpha generators being dominant in the deeper neocortical layers and the fact that occipital lobes are rarely covered by electrodes in human patients.

### 2.5 The alpha rhythm beyond the visual cortex

As the EEG recording equipment gradually improved it became possible to use a larger number of electrodes. This allowed for considering the spatial distribution of the alpha oscillations as well as how they were modulated in different regions given the tasks performed (42,43). When inspecting the topography of the alpha oscillations it became clear that they were not only produced in posterior brain regions, but evidence was also found for generators in sensorimotor areas (44,45). The central rhythm was termed the mu-rhythm (or the wicket rhythm), and it was blocked by engaging the somatosensory or motor areas. As such, it could be considered to have the same function as the alpha rhythm in the sensorimotor system.

The development of faster computers with more storage capacity allowed for improved signal processing in the spectral domain (46). The improved EEG equipment and automatized computer-based analysis also allowed for increasing the number of participants and thus the reliability of the reported results. This was first done using a measure termed event-related synchronization (ERS) and desynchronization (ERD) (47,48). These tools were based on bandpass filtering the signal in a specific frequency band and then averaging the time-development of signal magnitude over trials (47,48). The measures of ERD and ERS allowed for quantifying the modulations of the alpha rhythm in cognitive tasks. Extensive work was done to uncover how alpha-band oscillations were modulated in sensorimotor tasks (49) and it was demonstrated that modulations of the alpha-band mu rhythm could be distinguished from the hand versus foot areas (50,51). These findings suggested that the alpha rhythm might be operating on a much more precise spatial scale in the sensorimotor cortex as previously appreciated. Consistent with this, later EEG and MEG work demonstrated that alpha oscillations in the visual system are modulated to reflect the allocation of spatial attention in a retinotopic manner (52–55).

The ERD/ERS approach was also applied to EEG data from cognitive tasks in pioneering work by the group headed by Wolfgang Klimesch at the University of Salzburg. Semantic memory was investigated by judging whether two sequentially presented words were congruent in terms of their meaning (56,57). Alpha desynchronisation (in the upper-frequency band typically in the 10-12 Hz range) was related to semantic memory whereas episodic memory was related to modulations in the theta-band. While the relationship between episodic memory and theta oscillations has been confirmed by other studies, a causal link between alpha oscillations and semantic retrieval remains tentative. Later studies have shown robust modulations in the alpha rhythm in tasks where e.g. attention is modulated albeit the semantic elements are the same (58). Nevertheless, the Klimesch group pioneered cognitive investigations relating tasks-specific modulations in alpha oscillations to fundamental functions such as working memory, episodic memory and attention (59,60). This work formed the basis for uncovering the mechanistic role of alpha-band oscillations in cognitive tasks and inspired numerous studies in other groups.

The signal-processing tools for quantifying task-related modulations in brain rhythms were improved by using time-frequency representations of power calculated using sliding time-window approaches based on wavelets, Hilbert or Fourier transformations (61–63). These methods allowed for quantifying temporal developments of power over a broad range of frequencies. In conjunction with these signal-processing advancements, the number of electrodes used in investigations also increased. EEG systems with 64 electrodes became commercially available and some groups developed the use of systems with more than 100 electrodes (64). Another major improvement was the introduction of whole-head MEG with more than 100 sensors (65). MEG is based on sensitive magnetometers which measure the magnetic fields generated by intracellular dendritic currents synchronised across neurons. Unlike EEG, which measures the distribution of scalp potentials resulting from extracellular currents, the magnetic fields measured by MEG have less distributed spatial distribution and allow for better source modelling (66). For instance, using MEG and source modelling both somatotopy and retinotopy were identified in sensorimotor and visual areas based on modulations of the alpha rhythm (55,67).

In parallel with these developments, the field of human brain imaging was undergoing revolutionary developments with the advent of PET and fMRI (68). The possibilities provided by the new brain imaging tools fuelled a dramatic increase in cognitive neuroscience investigations. These developments further helped to evolve the theoretical framework associated with cognitive neuroscience and prompted EEG and MEG investigations on brain oscillations interpreted together with recent brain imaging results. The simultaneous acquisition of EEG and fMRI data made it possible to relate alpha oscillations to the BOLD signal (69–72). These factors set the stage for further investigations to uncover the functional role of brain oscillations in cognitive tasks.

### 2.6 The paradigm shift: alpha reflects inhibition rather than a state of rest

Until the turn of the century, the alpha rhythm was considered to reflect a state of rest in visual and sensorimotor areas (49). This prevailing view promoted by Adrian and Matthews 1934 remained largely unchallenged, namely that a network not receiving input would start to spontaneously produce ∼10 Hz oscillations. When brain regions were engaged either by visual input or by attention-demanding tasks, the alpha oscillations would decrease; a phenomenon referred to as alpha-blocking. This view also implied that alpha oscillations were not under active control and therefore the resting-state notion of the alpha rhythm limited their importance when understanding cognitive processes in the brain. A few studies were not fully in line with the resting-state or idling notion of alpha oscillations. For instance, it was reported that the alpha rhythm in some cases could remain strong in attention-demanding tasks not relying on visual input (73,74) and it was also known that the allocation of attention to the auditory modality would not produce alpha-blocking *per se* (75). An EEG study found more parietal alpha power for tasks that did not require attention to the environment (e.g. mental arithmetic) compared to tasks that did (e.g. counting verbs or Mooney face detection) (76). These findings did not, however, provoke a rethinking of the functional role of alpha oscillations.

**Figure 2:**
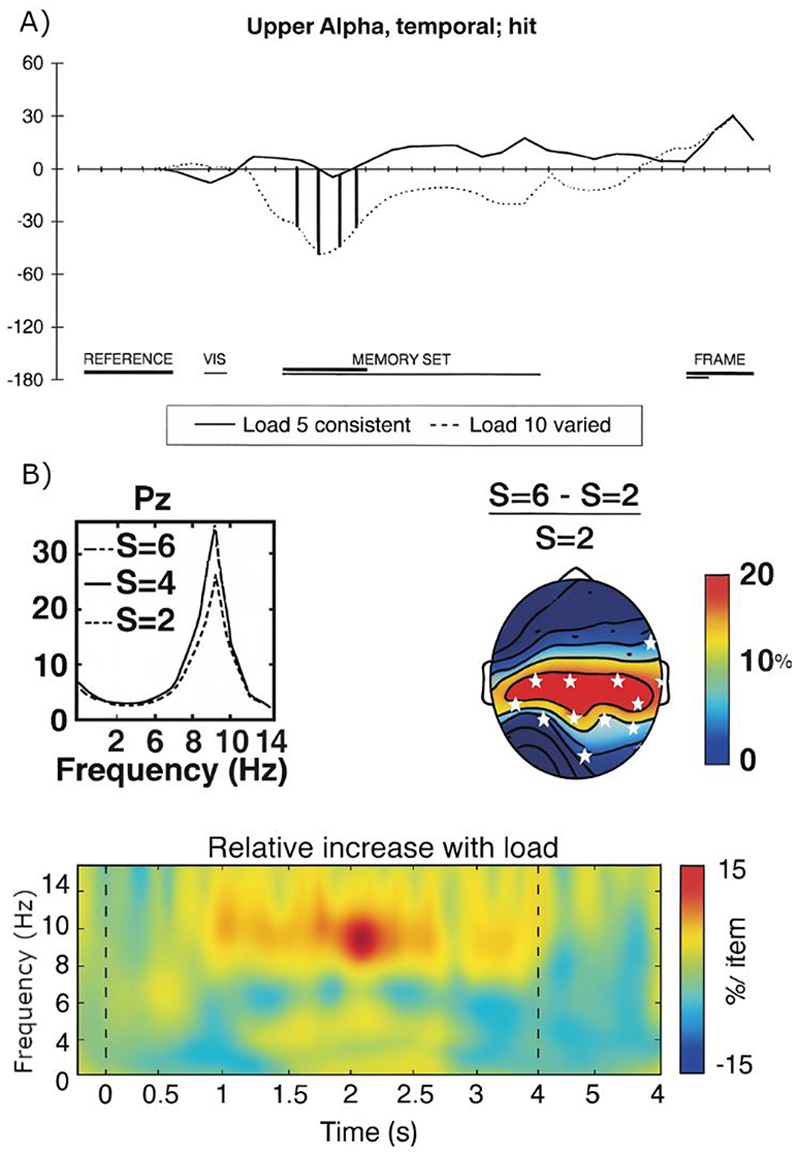
A) In a working memory study, alpha oscillations increased when 10 compared to 5 items were maintained in working memory. Here ERS and ERD denote respectively event-related synchronization and desynchronization (note that ERS is plotted negatively). Reproduced from (78). B) Using a Sternberg working memory task, a systematic increase in alpha power over posterior regions was reported. This increase in alpha power with memory load was sustained during the retention interval (from 0.2 to 3 s). Reproduced from (79);used with permission. These findings were best explained by posterior alpha oscillations actively inhibiting posterior brain regions during demanding tasks not requiring visual input. Consequently, they challenged the resting-state or idling notion of the alpha oscillations.

The real change in our understanding of the alpha rhythm occurred in the late 1990s prompted by several studies demonstrating that alpha oscillations increase during working memory tasks. This was first shown using an auditory working memory task in which posterior alpha oscillations increased during maintenance with respect to a baseline interval (77). In 1999 Klimesch reported on data from a working memory task involving the maintenance of 5 or 10 items (78). Surprisingly, the alpha power was stronger when 10 compared to 5 items were maintained. The authors interpreted this as resulting from inhibiting semantic long-term memory. The findings were corroborated by a study in which posterior alpha oscillations systematically increased with working memory load during maintenance (79) (Fig. 2).

Given the posterior topography of the alpha oscillations, the authors suggested that the alpha rhythm reflected functional inhibition of the visual stream. These conclusions were consistent with other observations on the allocation of attention. For instance, it was shown that alpha power increased over posterior areas when attention was allocated to the auditory as compared to the visual modality (80,81); these findings align with earlier reports by (75). In light of these findings, two interpretations of the increase in alpha oscillations could be entertained: 1) The alpha power increased as the visual system was left idle and computational resources were allocated to other regions or 2) the increase in alpha oscillations reflected top-down controlled inhibition of posterior regions which would serve to suppress interfering visual stimuli. To distinguish between these two possibilities, a MEG study was conducted in which anticipated interfering visual stimuli were presented during the maintenance of working memory (82). This study demonstrated better performance for trials in which the alpha power increased in anticipation of visual distractions. Similar results were obtained using EEG in a spatial working memory task (83). These findings provided strong support for alpha oscillations being inhibitory and under top-down control. In the case of working memory, the findings suggested that inhibition by alpha oscillations in task-irrelevant regions were required for optimal performance.

This collection of studies and theoretical considerations caused a paradigm shift in understanding the functional role of alpha oscillations as the idling notion of the alpha rhythm could not be maintained. The inhibitory properties of the alpha oscillations suggest that they play a functional role in which they inhibit areas not required for a given task to optimize performance. This new perspective further led to the suggestion that alpha oscillations might be serving a general role in gating the information flow in the brain by inhibiting task-irrelevant regions (84).

## 3 The physiological mechanisms responsible for generating alpha oscillations

While the functional significance of alpha oscillations has been extensively examined, our understanding of the specific neuronal mechanisms responsible for their generation remains incomplete. An intriguing explanation for this gap in understanding may lie in the disparity between the strong alpha oscillations observed at the scalp level in humans recorded by EEG and MEG and the challenges associated with detecting alpha oscillations in intracranial recordings. Another challenging aspect involves how a population of neurons collaborates to produce a rhythm with 100 ms cycles, especially considering that most physiological events, such as action potentials and synaptic communication occur on a 1 to 10 ms time scale. The understanding of the neurophysiological mechanisms generating the alpha rhythm has been derived using three approaches: direct electrophysiological recordings, pharmacological manipulations, and computational models. From the existing literature, it is evident that alpha oscillations may arise from intricate cortico-cortical and cortico-thalamic interactions. We will here summarise the brain regions and cortical layers in which alpha oscillations have been reported, identify the likely participating neuronal types and neurotransmitters, and examine various models employed to elucidate the mechanisms behind alpha oscillation generation. We focus particularly on the thalamus and neocortex associated with visual processing, as alpha oscillations have been studied extensively in these areas, though some findings regarding the somatosensory and motor systems are also considered.

### 3.1 Neuronal mechanisms contributing to the generation of the alpha rhythm

#### Pulsed inhibition

**Figure 3:**
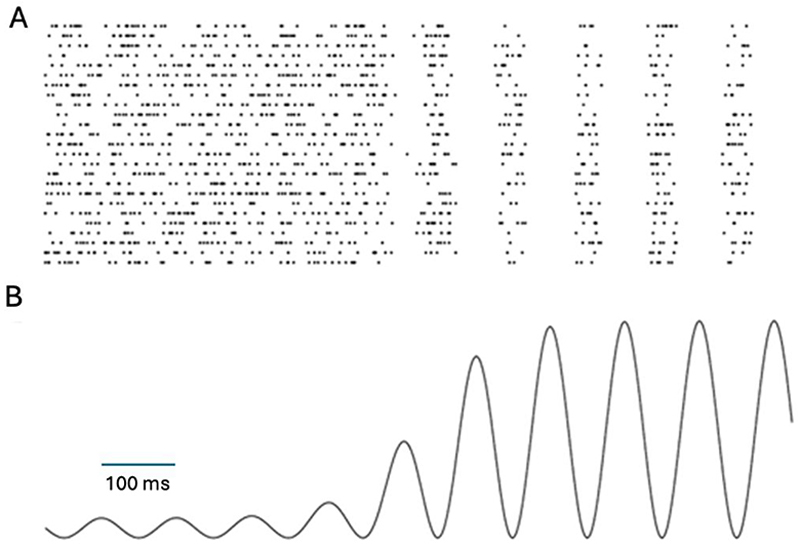
schematic explanation illustrating how alpha oscillations can emerge from a pulsed inhibition mechanism and there by explain the inverse relationship between firing rate and magnitude of the alpha oscillations. A) Consider a group of neurons firing as represented by the raster plot. Each line reflect one neuron. The neurons initially discharge at a high rate, but the firing is asynchronous (left). Eventually, the firing is inhibited in a pulsed manner every ∼100 ms, resulting in a population activity at ∼10 Hz (right). B). The population activity is measured as the local field potential, the EEG or MEG. Initially, no modulation is observed in the EEG as the firing is asynchronous (left). However, oscillations in the EEG are emerging due to the pulsed inhibition silencing the neurons periodically (right). This mechanism can explain the somewhat paradoxical finding of why alpha power in the EEG is inversely correlated with the firing rate. Adapted from (86). This scheme can also account for how alpha oscillations impact perception in an inhibitory and phasic manner (87).

When considering the mechanism generating alpha oscillations, one apparent conundrum is that how come the largest rhythm observed in the human EEG associated with inhibition of neuronal activity? This effect is best explained by the notion of pulsed inhibition (85,86). Consider a group of neurons all highly active but firing asynchronously. This scenario will not result in detectable modulations of the EEG or MEG signal at the scalp level. However, if the neuronal firing is interrupted every 100 ms due to pulses of GABAergic inhibition, a population signal will be generated emerging as a 10 Hz rhythm (Fig. 3). Importantly such a mechanism explains why neuronal firing is decreased as the alpha rhythm increases in power. Therefore, computational network models accounting for the emergence of the alpha rhythm should also work toward explaining how pulses of inhibition emerge from a mechanistic perspective.

#### Thalamic generators of the alpha rhythm

The thalamus, a deep brain structure with multiple nuclei, has been proposed to act as a pacemaker for the alpha rhythm (Lopes da Silva, 1991). The anatomical and computational complexity of the thalamus and its role in modulating neocortical interactions have more recently been considered in the light of oscillations and cognition (88). The alpha rhythm has been directly recorded in the thalamus in both humans and animals (89–92) and damage to the thalamus has been associated with a noticeable reduction in the amplitude of alpha oscillations (93). Several thalamic nuclei have been extensively studied in terms of their role in generating the alpha rhythm (94). The dorsal lateral geniculate nucleus (LGN), situated on the caudal inferior surface of the thalamus, receives input from the retina that it communicates to the primary visual cortex (90,95). It consists of relay cells that transmit sensory information to the neocortex, but they may also partake in the generation of alpha oscillations. The pulvinar is a higher-order thalamic nucleus that has also been implicated in modulating the alpha oscillations. Specifically, it may serve to synchronize extrastriate neocortical visual regions to coordinate the information flow (88). The reticular nucleus (RN) forms a capsule around the thalamus and does not project directly to the cortex, but rather it regulates neuronal activity within the thalamus. It is composed of a thin layer of inhibitory GABAergic neurons enveloping the thalamus and may be involved in modulating alpha oscillations (96).

**Figure 4:**
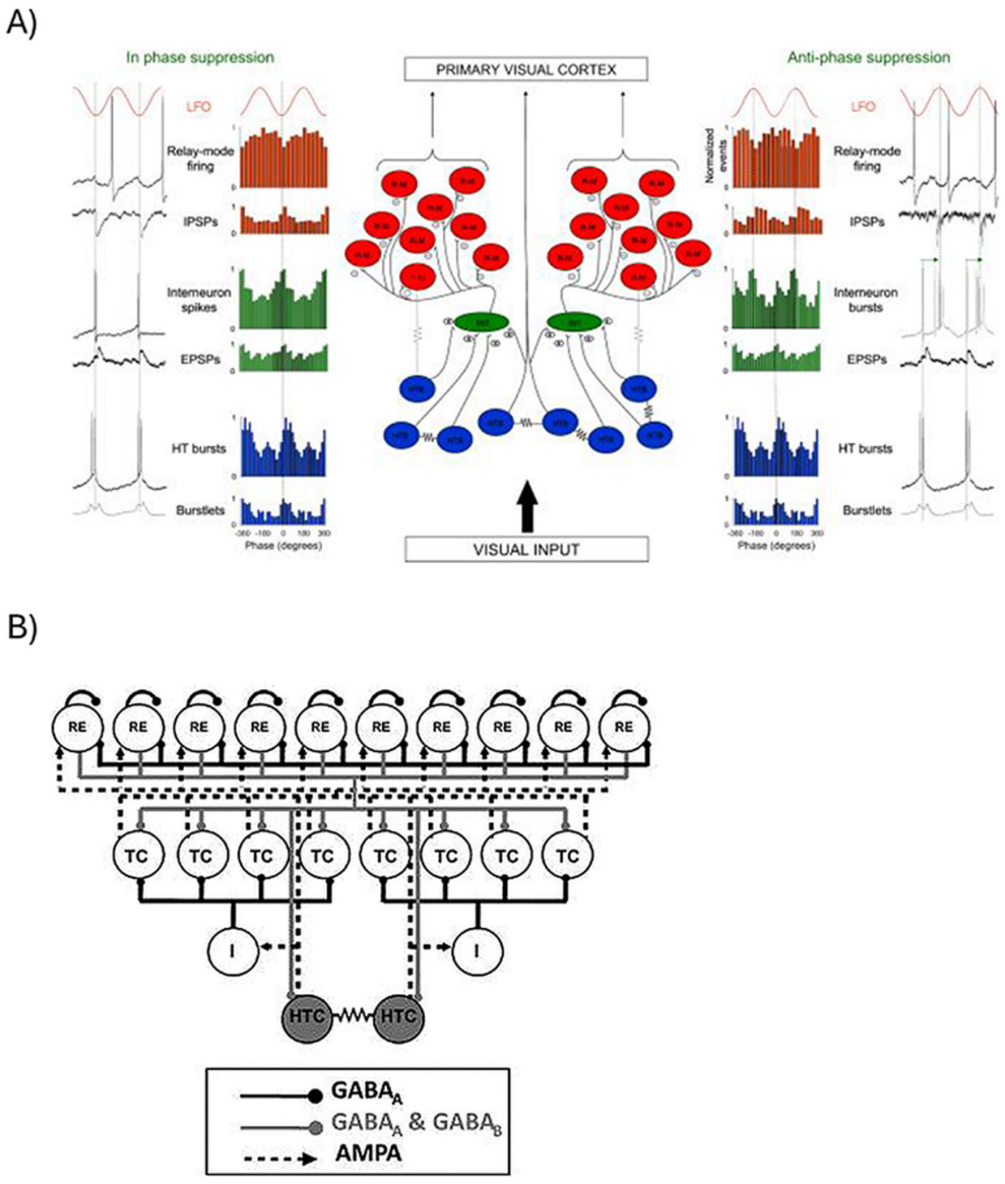
The thalamic mechanisms and regions involved in generating alpha oscillations. **(A)** Electrophysiology and pharmacology results from Lorincz et al. (2009). In the lateral geniculate nucleus (LGN), the activation of high-threshold bursting (HBT) neurons can lead to either spiking or bursting in interneurons (IN), which results in the suppression of relay-mode (R-M) neuronal activity at the LGN alpha peak or trough, respectively. The activation of muscarinic acetylcholine (ACh) receptors generates alpha oscillations and can induce HT burst firing in a subset of relay cells, producing phase shifts in their spiking. Reproduced from (92); used with permission. (B) The comprehensive computational model by Vijayan and Kopell (2012) reproduced both these physiological and pharmacological results. It further emphasised the effect of low-level activation of glutamate receptors, together with AcCh agonists, in initiating alpha activity that biases relay cells to fire at certain alpha phases. Reproduced from (151); used with permission.

Research focusing on the LGN has identified high-threshold (HT) bursting thalamocortical cells that shape rhythmic patterns and synchrony in the visual system. The bursts of these cells are supported by voltage-gated Ca^2+^ conductances (97,98). Consistent with a thalamic pacemaker role, gap junctions between HT bursting thalamocortical neurons contribute to the synchronisation of neuronal firing and thereby support the generation of the thalamic alpha rhythm (92,95,99,100). In awake cats, reverse microdialysis of gap-junction blockers in the LGN reversibly suppresses both LGN and EEG alpha and reduces local neuronal synchrony (101); strongly implicating electrical coupling in rhythmogenesis. Using a combination of *in vivo* recordings in awake cats and *in vitro* LGN–perigeniculate slice preparations, two classes of relay-mode thalamocortical neurons were identified that fire near either the peak or the trough of the alpha rhythm (92). For both groups of relay neurons, alpha-band timing arises through ∼10 Hz cyclic suppression imposed by LGN interneurons; when interneurons fire spikes or bursts, they produce, respectively, in-phase or anti-phase suppression of relay-mode neurons. These interneurons are driven by HT-bursting neurons receiving retinal inputs, such that relay throughput is temporally framed by phasic inhibition. This mechanism provides a concrete cellular implementation of pulsed inhibition that can gate visual information flow to the neocortex (Fig. 4). These thalamic mechanisms fit within a broader thalamo–cortical account in which LGN alpha generators couple with neocortical sources to organise large-scale excitability cycles and route information across the visual system (92,95,101–105). Evidence from humans also points to the LGN’s role: the individual frequency of occipital alpha correlates with microstructural properties of the optic radiation connecting the LGN and visual cortex (106).; conversely, recordings in non-human primates indicate that alpha can mediate feedback from neocortex to the LGN (107). The task-dependence of these bidirectional interactions warrants further investigation.

#### Neocortical generators of the alpha rhythm

While the alpha rhythm reflects the dominant spectral peak in human EEG and MEG recordings, reports based on intracranial recordings are more sporadic in both humans and non-human primates. In the early days, Adrian and Matthews did report one human case where alpha oscillations were observed intracranially (9) whereas a later review pointed to the difficulty in general of observing intracranial alpha oscillations (21). In non-human primates, it is only recently that reports on intracranial alpha oscillations started to emerge. The earlier absence of reports on alpha might be due to single-electrode recordings aiming to characterise spiking activity from individual neurons. These recordings would have missed alpha oscillations in the local-field potentials in deeper neocortical layers. Another issue pertains to how intracranial multi-electrode recordings are analysed in non-human primates. Some ECoG recordings use local references (e.g. deriving the first or second-order spatial gradient) and this might hinder detection given that the alpha activity is spatially phase-coherent (108). Nevertheless, a recent multi-electrode study in macaques reported strong oscillations in the alpha-band detected in multiple neocortical regions (109).

**Figure 5:**
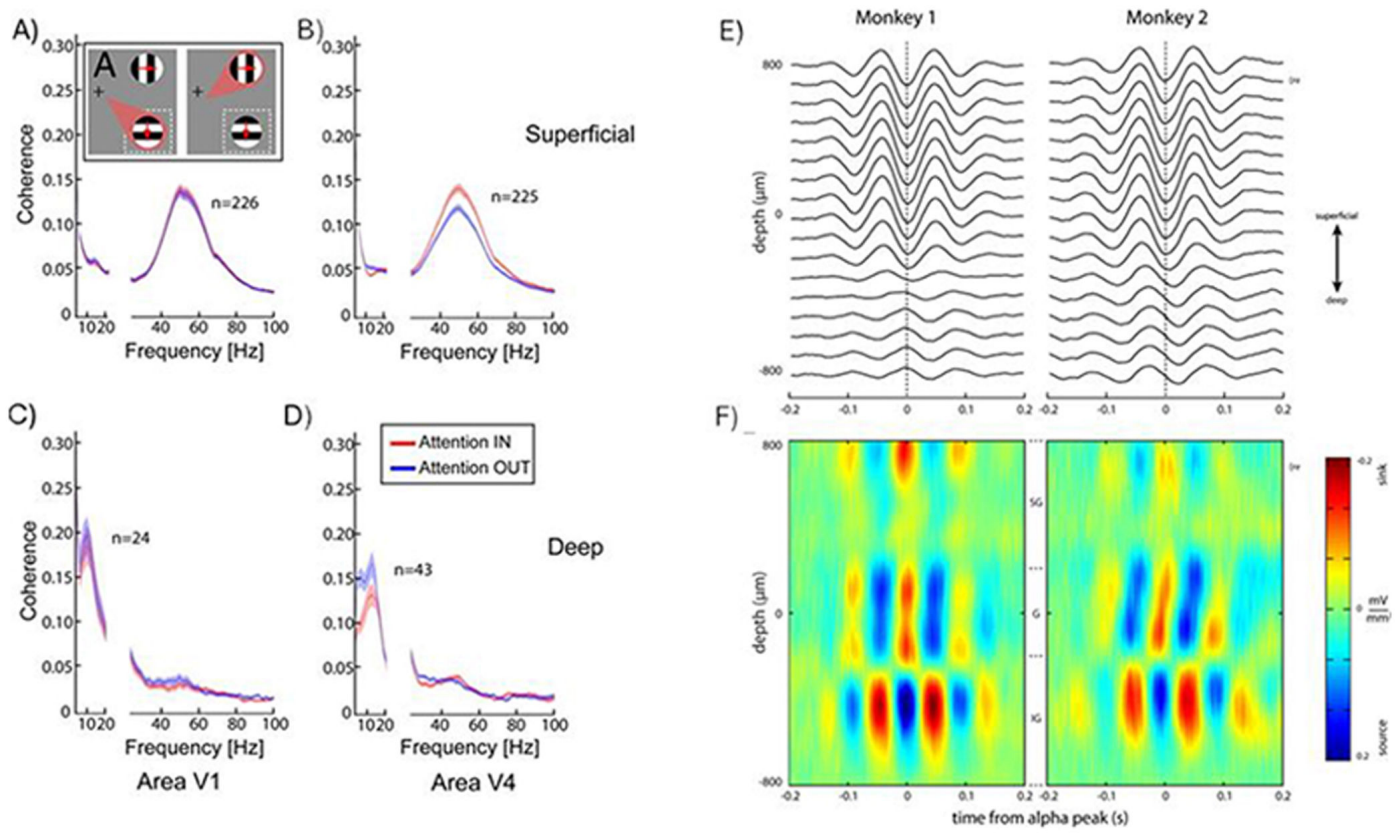
Experimental findings identifying deep cortical laminae sources of alpha oscillations. (A) Attentional modulation of spike–field coherence in areas V1 and V4, adapted from (35). Non-human primates were cued to attend to a moving grating either inside or outside the recorded neuron’s receptive field. Red and blue traces show spike–field coherence in each area when attention was directed into or out of the receptive field, respectively. Only gamma-band activity was detected. (B) In area V4, gamma coherence was strongest in superficial layers and increased with attention. (C) Alpha coherence was localised to deep layers, where gamma coherence was minimal. (D) In deep layers of V4, alpha coherence decreased with attention, whereas gamma coherence was not observed. (E) Laminar recordings in V1 of non-human primates in period of wakeful rest. The layers specific activity time-locked to alpha troughs: average voltage traces phase-locked to troughs identified in the superficial layers (reference electrode marked “(ref)”). (F) Current-source density maps corresponding to the traces in (E), showing alpha-frequency sink/source alternations in deep layers around −100 μm and −400 μm. Adapted from (36). See also (110) for similar findings. The deep layer generators of the alpha activity might explain why oscillations are seen so strong in EEG and MEG recordings as the deep layer activity would be associated with currents in the long dendrites of layer 5 and 6 pyramidal neurons.

Over the years the layer-specificity of the alpha generators has been extensively discussed. There is strong evidence for generators in deep cortical layers. This was first established by recordings in dogs (111) and later by recordings in non-human primates (35) (Fig. 5A-D). By analysing the current-source density profile of laminar recordings from V1 in non-human primates, alpha generators were confirmed to be in deep cortical layers (36). Even when signals from superficial layers were used to identify the phase of the alpha signals, the associated generators were strongest in deeper layers (Fig. 5E,F). The deep-layer alpha generators in the current-source density profiles observed in the laminar recordings are most likely explained by after-hyperpolarisation potentials following periodic bursting in layer 5/6 pyramidal cells. The after-hyperpolarisation potentials will result in currents flowing toward the cell bodies in the long parallel dendrites of the layer 5/6 cells. These dendritic currents would summate over a large number of parallel dendrites and could explain the relatively large signals in the alpha-band being measured at the scalp by EEG and MEG recordings. Nevertheless, there are also contributions to the layer 5/6 dendritic currents from synaptic input in superficial layers. Indeed sink-source profiles in the alpha-band have been identified in superficial layers (34,38,112–115). Specifically sink-source profiles in supragranular layers have been reported in non-human primates in several sensory areas (38). In human intracranial recordings in epileptic patients, supragranular alpha generators were also reported (115). These studies collectively suggest that alpha oscillations may play a functional role in most cortical layers, aligning with the idea that alpha oscillations reflect both neocortical feedback and input from the pulvinar engaging superficial and as well as deep neocortical layers (113,116,117). This framework has been further developed based on laminar recordings in non-human primates and resulted in the suggestion of spectrolaminar motifs across cortical regions in which superficial gamma activity reflects feedforward processing and deep layer alpha-beta activity reflects feedback (110) albeit the robustness of this framework has been questioned (118).

To summarize, there is evidence for both thalamic generators of the alpha rhythm in the LGN and the pulvinar as well as neocortical generators. The alpha oscillations measured by EEG and MEG are likely to be a direct consequence of neocortical generators. Furthermore, the large magnitude of the alpha oscillations at the scalp suggests they are generated by synchronized electrophysiological events that summate. Currents in the long and parallel dendrites of layer 5/6 cells meet this requirement albeit this does not exclude contributions from other cortical layers. Nevertheless, there remain many unknowns in terms of the interactions between cortical layers and thalamocortical interactions generating the alpha oscillations. Animal preparations employing optogenetic approaches to drive and inhibit neuronal activity in specific regions and layers hold great potential for further uncovering the functional role of alpha oscillations (119). For instance, by applying optogenetic rhythm stimulation in the thalamus of the ferret, it was recently shown that the higher-order thalamus coordinates both cortico-cortical and thalamocortical connectivity in a sustained attention task (120). These studies hold the promise of providing further causal insight into the role of the alpha rhythm, albeit the application is typically constrained to non-primates.

### 3.2 The role of neurotransmitters and modulators in the generation of alpha rhythms

As alpha rhythms have been linked to functional inhibition, one might think their generation would involve γ-Aminobutyric acid (GABA), an inhibitory neurotransmitter. Specifically, it has been investigated how GABA_A_ agonists, which enhance GABAergic transition, impact the generation of the alpha rhythm. While some studies using benzodiazepine, a GABA_A_ agonist, noted a decrease in posterior alpha during rest and tasks (121,122) other studies reported no effect (123) or even an increase (124). These discrepancies are somewhat surprising as benzodiazepines result in a robust increase of beta oscillations (123,125,126). As such the role of GABAergic interneurons in generating alpha oscillations is not well understood. Intriguingly, a combined PET-EEG study administrating Lorazepam (a benzodiazepine) compared to placebo controls, showed that EEG alpha oscillations were correlated with glucose metabolism in the bilateral thalamus and occipito-parietal areas during placebo administration. With the administration of lorazepam, alpha oscillations were reduced and the correlation between metabolism and alpha disappeared in cortical areas. These findings provide insight into the role of the thalamus in modulating the generation of the neocortical alpha rhythm (121). Propofol is a GABAergic agonist that impacts the alpha oscillations as well. When given in higher doses it will result in loss of consciousness and the generation of alpha oscillations over frontal areas (127). The effect has been explained by a computational model suggesting that propofol disrupts the normal alpha oscillations in posterior-projecting thalamic nuclei while engaging alpha generators in frontothalamic nuclei (128); see also (129).

Pharmacological interventions that act on glutamatergic excitatory transmission including AMPA, NMDA and metabotropic receptors, have been shown to modulate the generation of alpha oscillations. Using slice preparations of the somatosensory rat cortex, it was found that synchronized rhythmic activity in the alpha-band increased in layer 5 cells when NMDA conductivity was facilitated by reducing the concentration of extracellular Mg^2+^ (130). Within the LGN in cats, it was demonstrated that activation of the metabotropic glutamate receptor mGluR1a providing a tonic excitatory drive increased the frequency and reduced the amplitude of neuronal oscillations between 2 and 13 Hz (95). According to Lörincz et al. (2009), the excitatory glutamatergic drive from HT cells onto interneurons is essential for the temporal framing of relay-mode neurons in the LGN. In the visual cortex in non-human primates, it was found that AMPA and NMDA blockers consistently suppressed alpha power in V1 (113). In humans, a sub-anaesthetic dose of ketamine (an NMDA antagonist) were associated with a decrease in both parieto-occipital alpha power and frequency during rest but not during visual stimulation (131–135). In sum, as most neuronal interactions involve communication via excitatory and inhibitory synapses, any modulation in synaptic efficacy is bound to impact the generation of the alpha oscillations. Surprisingly, reducing the GABAergic transmission has resulted in ambiguous findings, while reducing glutamatergic excitatory transmission typically suppress the alpha oscillations.

Acetylcholine (ACh), which can act as both a neurotransmitter in the muscles and neuromodulator in the brain, has been shown to be linked to the modulation of alpha oscillations. The release of ACh is controlled amongst others by the medial septum, the pedunculopontine nucleus, and the basal nucleus of Meynert, projecting to various locations in the thalamus and cortex. ACh can bind to muscarinic and nicotinic receptors. The effects of agonist and antagonist drugs on muscarinic and nicotinic receptors have been reviewed in detail in (122). Specifically, drugs that block ACh transmission result in a decrease of the alpha oscillations, whereas increasing the efficacy of the ACh produces an increase. Such effects have been observed in human parieto-occipital alpha oscillations during rest (136) and spatial-attention tasks (137). Electrophysiological research done in cats revealed that alpha rhythms in the LGN require the activation of muscarinic ACh receptors (100). This work also demonstrated that muscarinic receptors play a critical role in inducing high threshold bursting in a subset of TC cells, with some TC cells shifting the phase of firing with respect to the alpha oscillations in response to muscarinic modulation. As such, ACh plays a role in modulating and possibly also generating the alpha rhythm.

Finally, drugs enhancing the effect of serotonin, a neurotransmitter widely distributed in the brain and often under the control of the Raphe nuclei, have mostly been associated with a significant decrease in posterior alpha power or an increase in frequency during rest and tasks (138–141). The relationship between serotonergic-modulating drugs, such as LSD and the impact on perceptual changes has been discussed (142) In particular, a link between visual trailing with various serotonergic drugs were considered in relation to oscillatory activity.

Dopamine is another important neurotransmitter acting throughout the brain. Dopamine has amongst others been associated with motor control and the rewards system and imbalances in dopaminergic modulation has been linked to schizophrenia, Parkinson’s Disease and ADHD. Various studies have investigated the impact of dopaminergic drugs on alpha oscillations. When administrating L-Dopa in patients with Parkinson’s Disease it results in a dose-dependent increase of the posterior alpha rhythm (143). Stimulants like amphetamine and cocaine also act on the dopaminergic system. Hans Berger found that cocaine increased the power of spontaneous alpha and beta oscillations (144), however later studies mainly point to an effect in the beta-band (145). These findings are contrasted by studies reporting a reduction in alpha oscillations with dexamphetamine (146). While it is clear that the dopaminergic system impacts alpha oscillations either directly or indirectly, the mechanism of action is not clear.

In sum, it is clear that both glutamatergic and GABAergic transmission impact the generation of alpha oscillations, and they are generated by interacting networks of excitatory and inhibitory neurons. Perhaps surprisingly, while most neuromodulators have been reported to impact the magnitude of the alpha oscillations there is not a clear picture emerging of some being more important than others. This suggests that the effects of neuromodulators are primarily indirect.

### 3.3 Computational models of alpha rhythm generation

Insight into the neuronal mechanisms underlying the generation of alpha oscillations has been investigated by modelling studies, which have primarily focused on the interactions between the thalamus and cortex. Although no computational models have fully addressed the complexity of the alpha rhythm, including the directional flow, frequency specificity, and laminar specificity, they have offered insights into some of the parameters that are particularly important for generating the alpha rhythm. One of the key questions these models must address is how the ∼100 ms period of the alpha rhythm is generated in the light of the observation that the time course of most synaptic interactions occurs on a <10-20 ms time scale, i.e. which mechanisms serve to bridge the 100 ms and determine the frequency of the oscillations?

Computational work in the 70s laid the groundwork for understanding how alpha rhythms are generated in neocortical networks interacting with the thalamus (147). This model was constrained by histological and biophysical data including animal recordings. The model included two populations of neurons interconnected by means of inhibitory interactions. The model showed that when the network was driven by trains of pulses with a Poisson distribution, alpha oscillations were generated in the population of neurons; as such the model has resonance properties in the alpha-band; a property indirectly confirmed later by EEG and broad-band visual flicker (148). However, this dynamic was achieved by having time constants of inhibitory interactions spanning ∼100 ms which is somewhat physiologically unrealistic considering the time constant of GABAergic interactions being much faster.

#### Detailed physiologically constrained models

Subsequent models have been developed to incorporate the ion-channel dynamics of neocortical networks. A model developed to account for ∼10Hz oscillation in somatosensory cortex focused on the properties of somatosensory layer 5 inhibitory and pyramidal neurons considered the dynamics contributed by hyperpolarisation-activated currents (I_h_) and low-threshold calcium currents (I_T_) membrane currents. These receptors together produced rebound excitation in the pyramidal neurons following the GABAergic inhibition from interneurons. The time course of the rebound was at a time scale of 100 ms thereby explaining the emergence of a rhythm in the alpha-band (149).

In the visual cortex, the specific role of glutamate receptors for generating the alpha rhythm was recently investigated. This model aimed to account for the post-stimulus alpha rhythm, emerging after bouts of gamma oscillations in layer 4 of V1 networks (150). According to this model, alpha oscillations are generated by an interaction between a subtype of NMDA receptors which do not have magnesium-dependent receptor blocking as well as subthreshold potassium receptors. The alpha rhythm emerges from the interaction between the de- and hyperpolarisation provided by respectively the NMDA and the potassium receptors. While these models provide physiological accounts for the generation of the alpha rhythm considering the receptor kinetics, further work is required to determine whether the proposed mechanism in general can explain the human alpha rhythm.

Other models have focussed on the generation of the alpha rhythm in thalamus. The work of (151) developed a detailed conductance-based model to account for alpha oscillations in a network comprising thalamic reticular (RE), thalamocortical (TC), and high-threshold thalamocortical (HTC) cells (see Fig. 4B). Key to the rhythm generation was the activation of either muscarinic acetylcholine receptors (mAChR) or metabotropic glutamate receptors 1 (mGluR1). With activation of mGluR1 receptors TC cells fire during any phase of the alpha cycle, while when mAChR were activated, TC cells fired at the peak or the trough of the alpha oscillations. This behaviour is in line with data recorded in cats presented by Lörincz et al. (2009): HTC cells in this model provided excitation to interneurons that inhibit TC cells, while HTC and TC cells excited RE cells that inhibited them via GABAergic inhibition (Fig. 4A). The model can further account for the finding that low levels of mGluR1 activation combined with mAChR agonists may be able to initiate alpha activity that biases TC cells to fire at certain phases of the alpha rhythm. This mechanism offers a pathway for control of cortical neuronal activity. In sum, this model provides insight into how the brain processes or blocks sensory information based on glutamatergic increases on alpha power. While this model framework is supported by animal data, it remains to be determined whether the mechanisms generalise to human alpha oscillations.

#### Neural mass models

Neural mass models have also been developed which incorporate less physiological details and do not model spiking activity *per se*. These mean field models allow for simulating the averaged activity of ensembles of neurons at a more abstract level while incorporating some realistic biophysical parameters. Many of these models are anchored in the seminal work of Lopes da Silva (Lopes da Silva et al., 1974) and have been developed to account for the global dynamics produced by a larger network to explain the emergence of brain oscillations and evoked responses. The models have been extended to involve multiple alpha rhythm generators organised in visual cortical columns. This class of models can account for spontaneous alpha rhythms, stimulus-locked alpha oscillations as well as input driven decreases and increases of alpha power. Specifically, the stimulus-induced changes in power at the population level can be explained by coupling and decoupling of multiple cortical columns in the alpha-band (152).

The interaction between different networks of the thalamus and the neocortex has also been simulated using neural mass models (153). This work makes the case that alpha oscillations primarily result from corticothalamic feedback resonances. Other related work has considered the role of the thalamus for resolving competition at the neocortical level (96). The model could account for earlier EEG studies reporting a decrease in alpha power over the central sulcus surrounded by alpha power increases following movements and somatosensory stimulation (154). The simulation studies revealed that this antagonistic phenomenon would depend on the interactions between populations of thalamocortical and reticular nucleus cells. Within the reticular nucleus, the interactions between the different sections that correspond to different sensory information (e.g., hand, foot) are crucial for the competition in the alpha-band to emerge at the cortical level.

Mean field models have also been developed to explain the interaction between alpha oscillations and neuronal activity reflected by firing and BOLD activity. A thalamocortical model was developed to account for the inverse relationship between alpha power and neuronal activity (155). This work is part of a larger initiative with the aim of making large-scale models of brain dynamics (156). Finally the neuronal mass models can also be constructed to produce changes in the alpha rhythm as for instance observed in dementia patients (157). This work suggested that modifying synaptic connectivity in the thalamus altered alpha-band power and frequency consistent with changes observed in patients with Alzheimer’s Disease.

#### Summary on physiological models

In sum, many models have been constructed to account for the emergence of the alpha rhythm. While each of these models has interesting elements, they have not resulted in coherent framework providing a unified account on the generation of the alpha rhythm. While there are multiple candidates for the physiological mechanism responsible for generating the ∼100 ms periodic activity accounting for the frequency of the alpha rhythm, no consensus has yet been reached. There are also diverging accounts on the importance of the thalamus for generating the rhythm; in some models the alpha rhythm can be generated in neocortex while modulated by the thalamus, whereas in other models the thalamic drive is essential. In future work it would be important to conduct studies aim to more precisely identifying the precise neuronal mechanisms determining the frequency and network properties of the alpha rhythm.

### 3.4 The networks exercising top-down control of the alpha rhythm

Large scale analysis of EEG and MEG data have demonstrated an intricate network of generators of the alpha oscillations (158,159). Some of these generators might exert control on others depending on task context. Specifically, parieto-occipital alpha power is modulated during tasks involving working memory as well as the allocation of attention which demonstrate that these oscillations are under top-down regulation. We discuss the control network involved in modulating posterior alpha oscillations.

#### Frontal eye-fields and dorsolateral prefrontal cortex

Studies based on intracranial recordings in macaques as well as human imaging have highlighted the role of the dorsal attention network in the allocation of attention. Particularly the FEF has been associated with spatial attention. This notion is supported by research in macaques demonstrating the existence of direct anatomical projections from the FEF to parietal and visual areas including V1 and V4 (Barone et al., 2000; Markov and Kennedy, 2013). Numerous studies have uncovered the role of the FEF in modulating parieto-occipital alpha oscillations both in terms of magnitude and phase in spatial attention tasks (117,160–165). For example, an MEG study found that the right FEF controls posterior alpha during a covert spatial attention task, as evidenced by measures of Granger causality (165). Moreover, the anatomical connection strength between the FEF and posterior areas detected as the superior longitudinal fasciculus correlates with in the individual ability to modulate both alpha and gamma power in a simple spatial attention task (166). Even more striking evidence comes from studies using repetitive transcranial magnetic stimulation (rTMS) to disrupt FEF activity during attention tasks. These studies have shown alterations in anticipatory posterior alpha modulation, measured with MEG or EEG, suggesting a direct influence of FEF on alpha dynamics (163,167). Using brain stimulation to infer a causal relationship, it was demonstrated that repetitive TMS over FEF in one hemisphere was associated with a reduced ability to modulate alpha power in contralateral posterior regions (163). This somewhat surprising inter-hemispheric interaction has been suggested to be explained by a mechanism in which engaging one hemisphere results in an increase in alpha oscillations inhibiting the other hemisphere (168,169). Beyond FEF, several studies have also reported the dorsolateral prefrontal cortex to modulate posterior alpha oscillations in paradigms involving feature and cross-modality attention tasks (82,164,170,171). Finally, multi-unit recordings have also been performed in the frontal-eye fields of non-human primates (172). This work revealed that the attentional spotlight could be decoded from the neuronal activity and that exploration of space was clocked by a 7-12 Hz alpha rhythm. In future work it would be interesting to directly link FEF activity related to covert exploration to alpha oscillations in posterior brain regions.

In short, brain regions associated with executive control are involved in the modulation of posterior alpha oscillations. In particular, the FEF seems to exercise causal control of posterior alpha oscillations likely via the superior longitudinal fasciculus. Evidently the FEF does not operate alone but rather serves as an important node in the control network.

#### The pulvinar nucleus mediating top-down control

As previously discussed, alpha oscillations have long been known to be detectable in the pulvinar (102,173,174) and this opens the possibility that the pulvinar is part of the network controlling neocortical alpha oscillations. The pulvinar is a complex region and the largest of the thalamic nuclei in humans (see (175)). The ventro-lateral pulvinar is highly interconnected with the different parts of the visual hierarchy in the ventral stream, the superior colliculus, and other sensory and association areas of the cortex. It also interacts with the reticular nucleus enveloping the thalamus. Both lesion studies and electrophysiological recordings have implicated the pulvinar in the modulation of cortical alpha oscillations, in particular, by controlling inter-cortical synchronization and thereby regulating the information flow (104,176–179). Studies on deactivations of the pulvinar in non-human primate resulted in an increase of neocortical alpha oscillations (176). This suggests that the pulvinar is not essential for generating the oscillations, but that it plays a modulatory role.

Specifically, the pulvinar was shown to drive the synchronization in the alpha-band between V4 and TEO when macaques allocated attention to a visual target (104). The degree of synchrony determined the functional connectivity reflected in the gamma-band. In sum, these findings speak to the importance of subcortical regions and in particular the pulvinar in modulating neocortical oscillations to support cognitive tasks.

### 3.5 The relationship between alpha and oscillations in other frequency bands

#### The coupling between alpha and gamma oscillations

If alpha oscillations are under top-down control and associated with functional inhibition, it leads to the prediction that they interact with gamma-band activity. The gamma activity is typically associated neuronal excitability and feed-forward processing. Indeed, the phase of alpha oscillations has been found to modulate gamma activity in resting-state MEG data—a finding confirmed by ECoG recordings (180). In macaques, laminar recordings from V1 revealed strong coupling between the phase of alpha oscillations and gamma power (36). Specifically, the magnitude of deep-layer alpha oscillations and superficial-layer gamma bursts were found to be anticorrelated. This aligns with optogenetic studies showing that deep-layer neurons suppress superficial-layer neuronal activity through intercolumnar inhibitory connections (181). ECoG recordings further indicate that alpha oscillations often manifest as travelling waves, with gamma-band activity coupled to the phase of these waves, effectively “surfing” across cortical areas (182). Task-dependent modulation of alpha-gamma coupling has also been observed in spatial attention and memory tasks. For example, in an MEG study, stronger pre-stimulus alpha suppression predicted enhanced gamma oscillations and better encoding of visually presented items into long-term memory (183) . Similarly, in a working memory task, pre-distractor gamma power coupling to alpha phase was associated with the ability to suppress visual distractors (184). In a recent study using intracranial data in humans, a coupling was found between the phase of the alpha oscillations and high-frequency gamma power during the allocation of spatial attention (185). Measures of directionality allowed for further uncovering when the alpha oscillations were controlling the gamma power and *vice versa*. Complementary findings using MEG showed similar alpha-gamma coupling using the same task as in the aforementioned intracranial study (165). Importantly, the directional coupling between alpha and gamma oscillations has clinical relevance. For instance, in a visual detection task, feedforward connectivity (V1-to-V4) was mediated by gamma oscillations, while feedback connectivity was mediated by alpha oscillations. The latter was significantly reduced in individuals with autism spectrum disorder (186). In short, there are numerous studies pointing to a coupling between alpha and gamma oscillations in terms of power-to-power and phase-to-power interactions. This coupling is often antagonistic, i.e. stronger alpha power correlate with a reduced gamma power in a pulsed inhibitory manner. While some work has pointed out that phase-to-power coupling in some case could be confounded by higher harmonics of non-sinusoidal alpha oscillations (187) subsequent work has defined the criteria for robust coupling and made a strong case for alpha phase to gamma power coupling (188,189)

#### Do alpha and beta rhythms reflect similar functional roles?

Many studies have reported a decrease of alpha (also referred to as the mu rhythm in the sensorimotor system) and beta-band oscillations during anticipation and stimulus processing in visual, somatosensory and motor areas (48,190–193). Although, in some cases, the beta power might reflect the higher harmonics of alpha oscillations, many experiments have shown that alpha and beta oscillations can operate independently (159,194,195). Indeed, recent work suggests that beta power modulation is reflected by changes in occurrences of bursts rather than sustained oscillations being up or down-regulated (196–199). Although these alpha and beta rhythms typically are associated with functional inhibition, there are differences. For instance, beta bursts have been related to short-lived stop signals in a no-go task while the mu rhythm (the alpha-band) has been associated with sustained inhibition of automatic responses (199,200). Another dissociation is that GABAergic agonists robustly increase the beta power in the somatosensory system (123,126,201) while they typically decrease or do not alter alpha power over posterior regions.

#### The relationship to spindles

Spindles are rapid bursts of brain activity typically observed during stage 2 of non-REM sleep and have been associated with memory consolidation (202).They occur during sleep in the 11-16 Hz frequency range and have sometimes been considered functionally similar to alpha oscillations. However, sleep spindles, unlike alpha oscillations, are generated in the TRN which receives excitatory input from thalamocortical neurons which project to the thalamus via inhibitory connections. This creates a feedback loop generating the oscillatory patterns characteristic of spindles (203). The generation involves T-type calcium channels within the TRN. While spindles initially were thought to be related to alpha oscillations, a strong case was made that barbiturate-induced spindle activity and the classical alpha rhythm are different physiological phenomena (33,90). Notably, the barbiturate-induced spindle activity is topographically more widespread than the posterior alpha and the thalamocortical coherence is stronger (33).

## 4 Alpha oscillations and cognition

As mentioned earlier, the alpha oscillations were until the ∼2000s considered an idling or resting-state rhythm. The view was challenged by the observation that alpha oscillations remained strong during working memory retention resulting in the notion that they reflect functional inhibition of visual regions (78,79). This inhibition likely serves to suppress potential distracting information thus allocating neuro-computational resources to the task at hand. This principle generalizes beyond working memory task, and the functional role of alpha oscillations has thus been investigated in a large set of cognitive tasks. Here, we discuss tasks in which alpha modulation is robustly observed, ranging from basic perceptual paradigms to language comprehension. Given the vast literature, we cannot cover all domains; howevernthe collection of studies will underscore that the primary role of alpha oscillations is to serve the allocation of computational resources within brain networks by suppressing tasks-irrelevant regions through pulsed inhibition.

### 4.1 The modulation of perception by alpha oscillations

Given the inhibitory role of alpha oscillations, perception should be modulated by the magnitude of the oscillations. Several MEG and EEG studies in which near-threshold stimuli were presented have confirmed this prediction. Essentially trials in which the posterior alpha oscillations were higher predicted a reduced ability to detect the stimuli (204–207). More specifically, some studies have reported an influence of amplitude on both the probability of reporting near-threshold stimuli and of reporting the presence of a stimulus (208,209). These findings associated alpha power to a change in the *criterion* as defined by signal detection theory. It has therefore been suggested that an alpha power decrease result in an increase in neuronal excitability thereby reducing the decision boundary for determining whether a stimulus is present (see Samaha et al., 2020).

Similar studies have been done in the somatosensory domain using MEG. Here, an inverted U-shaped relationship was found when relating single trial alpha power to detectability (211): intermediate amplitudes were associated with better detection whereas performance was reduced in trials with low and high alpha power. These findings raise the question of whether the neuronal excitability in sensory cortices is directly modulated by the magnitude of ongoing alpha oscillations. This has been investigated relating pre-stimulus oscillations to event-related potentials. The core finding was that both the C1 and the N150 are reduced with strong pre-stimulus alpha power in the visual domain (212). Since the C1 is thought to reflect afferent input to V1, this speaks to alpha oscillations playing a role in modulating the thalamic input to early visual cortex. This is also supported by intracranial recordings in non-human primates in which the increase in alpha-band was shown to correlate negatively with bursting in the gamma-band (36). Several studies have recorded the ongoing EEG while also measuring the BOLD signal using fMRI (69,213–216). These studies have consistently found that the BOLD signal in visual and sensorimotor cortices correlates negatively with the magnitude of the alpha oscillations. Finally, it has been demonstrated that the detection of phosphenes elicited by TMS pulses is reduced in phasic manner in trials when posterior alpha activity is strong (217). In sum, there is strong converging evidence using a range of experimental approaches demonstrating that increases in the alpha oscillations are associated with decreased neuronal excitability and perceptual detection abilities.

Another important question pertains to the phasic impact of the alpha oscillations. Several studies using difficult-to-detect visual stimuli have demonstrated that the phase of pre-stimulus alpha oscillations affect detection ability. Using visual paradigms in combination with EEG recordings, it was demonstrated that perception was modulated by the phase of the ongoing alpha oscillations (207,218). This resulted in the notion of *perceptual cycles* in which vision is sampled at the frequency of the alpha rhythm (87). This principle has been supported by findings demonstrating that the magnitude of the visually evoked BOLD response is modulated by the pre-stimulus phase of ongoing occipital alpha oscillations (191); similarly, the visually evoked C1 is also modulated by alpha phase (219). In support, intracranial recordings in non-human primates have demonstrated that the phase of ongoing alpha oscillations modulate both neuronal spiking and high-frequency gamma-band activity (35,36,184,188). Spaak et al. (2012a) and Bonnefond & Jensen (2015) further demonstrated that the negative correlation between alpha power and gamma power was specific to a particular phase of the alpha cycle. In sum, these findings make a strong case that alpha oscillations exert a phasic inhibitory drive on neuronal activity (see Fig. 5).

The notion of perceptual cycles also has consequences for how visual stimuli are processed and grouped in time. The core premise is that two items being presented slightly apart in time within the period of one alpha cycle will be integrated as compared to when they happen to appear in separate alpha cycles; a phenomenon referred to as perceptual framing (220). From the temporal framing hypothesis, it follows that for alpha oscillations with slower compared to faster frequencies, items presented slightly apart in time are more likely to be grouped. This prediction has been supported by EEG studies using various kinds of visual stimuli e.g. (221,222); however, another study has challenged how general these findings are (223). The debate is still ongoing and has been the subject of recent reviews reaching different conclusions on the reliability of temporal framing by alpha oscillations (224,225).

In conclusion, while there is converging evidence for a pulsed inhibitory role of alpha oscillations on perception, it remains debated whether these oscillations can support perceptual grouping.

### 4.2 Alpha oscillations and the allocation of spatial attention

The finding that alpha oscillations reflect region-specific inhibition and are under top-down control has resulted in the idea that they support the allocation of computational resources. This framework can be studied using attention tasks in which the alpha oscillations serve the purpose of reducing interference from irrelevant or distracting input.

**Figure 6:**
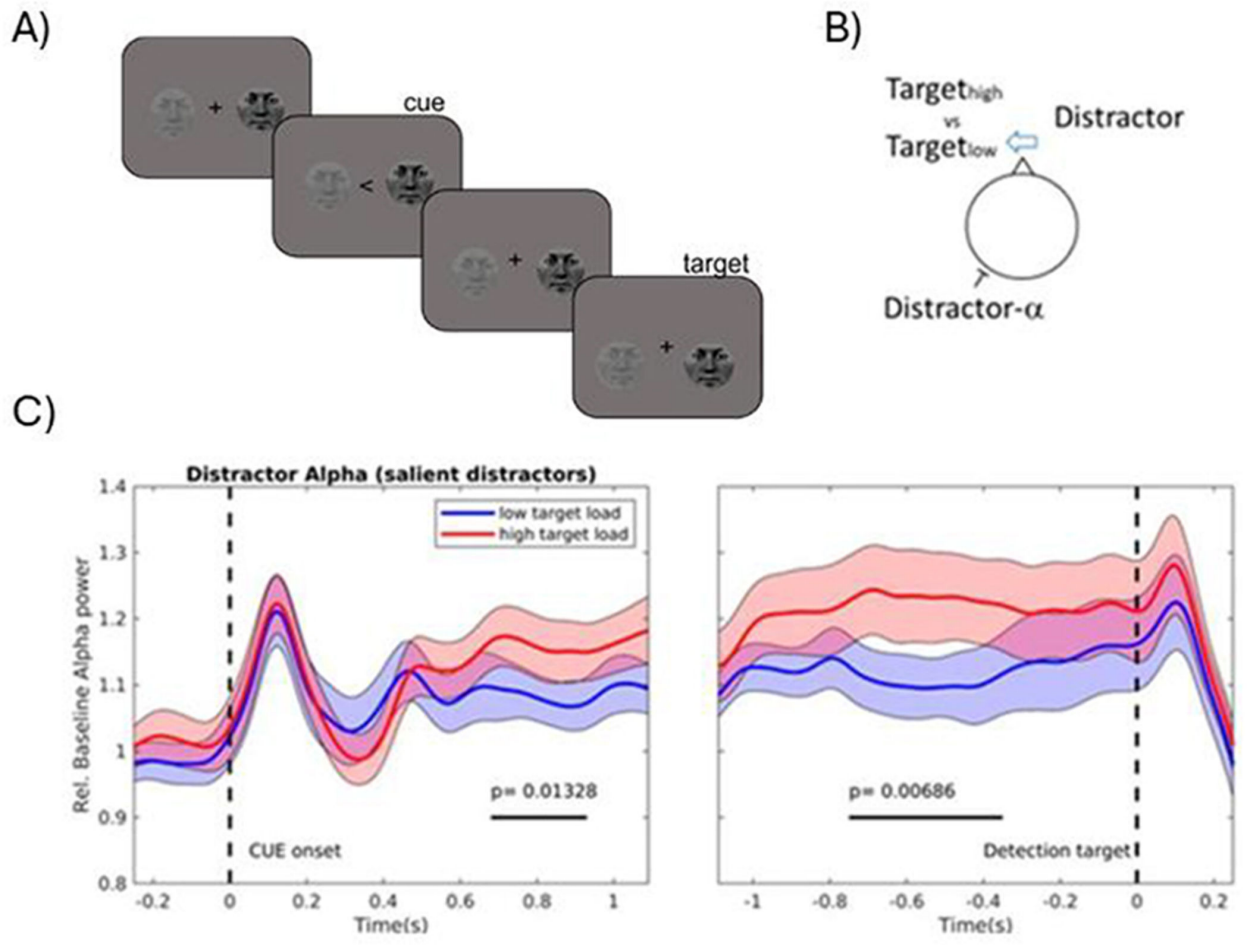
A) Attention task involving noisy and salient faces as target in one hemifield (targets with high and low perceptual loads respectively) and distractors in the other hemifield (noisy and salient distractors). B) In this example targets are presented to the right and distractors to the left. C) Alpha power contralateral to distractors was stronger in the high compared to low perceptual load condition. This difference in alpha power also correlated with the individual ability to ignore distracting stimuli. Reproduced from (236); used with permission.

Paradigms on spatial attention have become the workhorse for investigating the role of alpha oscillations at the network level. The first EEG study on spatial visual attention was conducted by (226). In this study, participants were cued to attend to stimuli appearing 1 s later in the right or the left visual hemifield. The core finding was a decrease in alpha power in posterior regions contralateral to the attended hemifield, while the alpha power increased ipsilaterally. These findings have been replicated in a large number of EEG and MEG studies (for a few examples see (227–230)). While alpha oscillations clearly are under top-down control, it has been debated to what extent they increase in anticipation of distracting stimuli (169,231–233). This question has been investigated in paradigms in which the likelihood of distractors in the left or the right hemifield was manipulated. Several studies failed to find alpha oscillation increases contralateral to the hemifield of the anticipated distracting stimuli (229,233), while other studies did find such a relation (234–236). The crux of the debate is whether distractors are suppressed by direct or indirect mechanisms; this is a general question going beyond the role of alpha oscillations (231). Indeed, *perceptual load theory* suggests that attention allocated to the target determines the degree of distractor suppression (237). In an MEG study it was explicitly tested whether the modulation of alpha oscillations was driven by the perceptual load of the targets or the degree of distraction. This was done by manipulating the perceptual load of targets and distractors in different hemifields (236). The core finding was that the perceptual load of the targets robustly predicted the increase in alpha power associated with the distractor (Fig. 6). Furthermore, this effect correlated with the individual ability to ignore distracting stimuli.

These findings are consistent with perceptual load theory in the sense that the distractor-related alpha power increase is primarily related to the perceptual load of the targets rather than the distractors. According to this framework, alpha oscillations are under top-down control, but the control is indirect in the sense that the increase in alpha power is determined by the allocation of resources towards the tasks at hand (169). In conclusion, a framework is emerging in which alpha oscillations clearly are involved in the allocation of computational resources in spatial attention tasks. Recent findings are converging on the notion that alpha oscillations are under indirect control by the regions to which resources are allocated (for a more general discussion on this debate, see (168).

**Figure 7:**
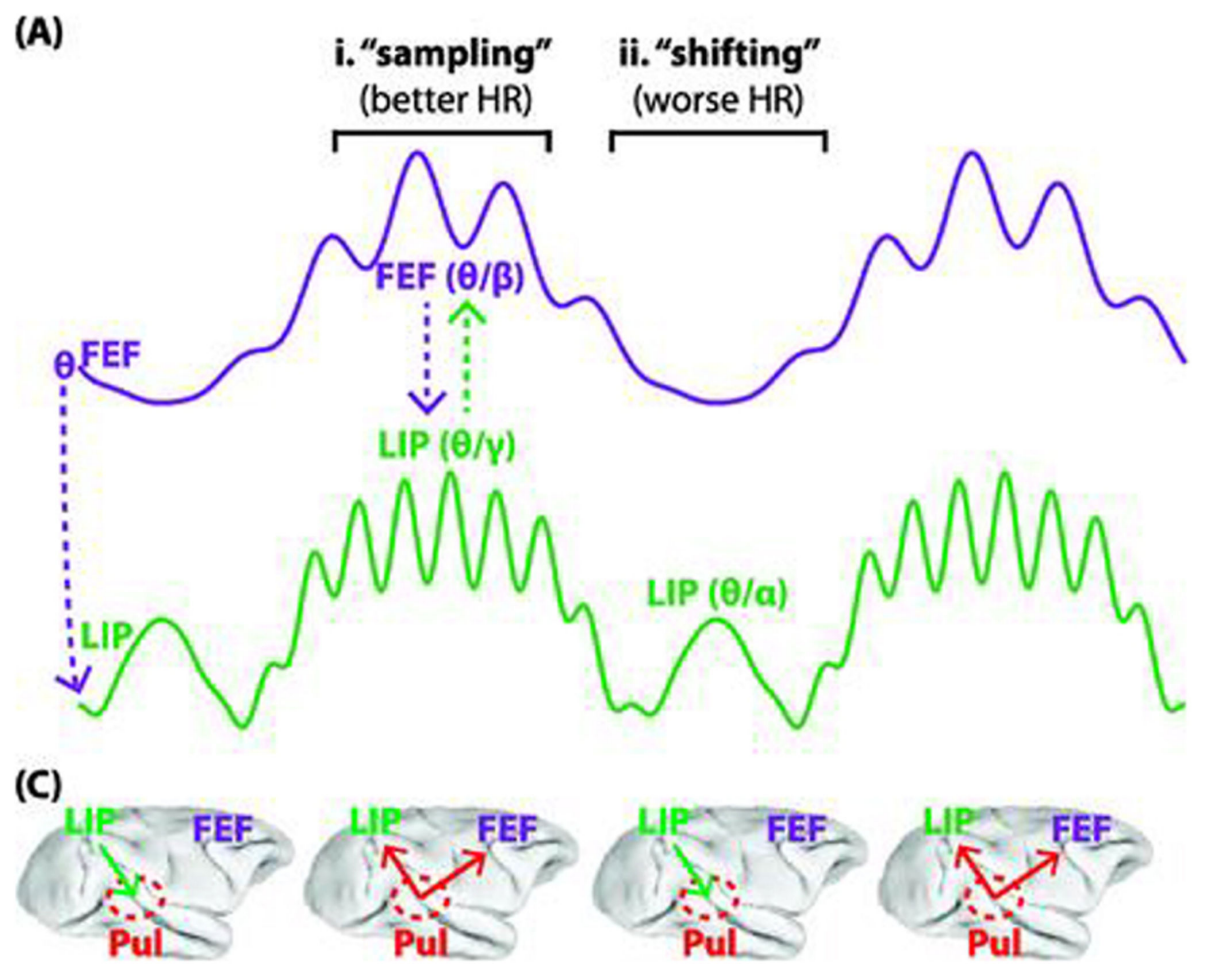
A schematic model of how oscillatory activity may coordinate the allocation of spatial visual attention. In the “sampling” state, elevated beta-band activity in the frontal-eye-field (FEF) corresponds with suppression of attentional shifts and/or saccadic eye-movements. Concurrently, increased gamma-band activity in the lateral intraparietal area (LIP) reflects enhanced sensory processing and improved behavioural detection at attended locations The pulvinar coordinates the neocortical activity. In contrast, the “shifting” state is marked by a rise in alpha-band activity in LIP, which corresponds with attenuated visual processing during which attention (covert or overt) may relocate to another spatial locus. In this case the LIP coordinates the activity in the pulvinar. The transition between these sampling and shifting states has been proposed to be paced by a theta-band rhythm (∼3-8 Hz), acting as a “clock” that alternately gates sensory-sampling versus attentional-relocation phases. Reproduced from (238); used with permission.

In the spatial attention tasks covered so far, the allocation of covert attention is driven by explicit cues. In real life situations the allocation of attention results from complex interactions between the visual input in a task context and intrinsic neuronal dynamics. It has been suggested that the allocation of attention is driven by a mechanism in which the shifts of allocation of attention fluctuates rhythmically. Indeed, several studies have provided behavioural support for the rhythmic allocation of attention governed by theta oscillations (239–241) albeit concerns have been raised on methodological aspect of these studies (242). These concerns do not pertain to electrophysiological studies linking neuronal activity to the dynamic allocation of attention in human and animals (177,241,243). Recordings from the FEF, the LIP, and the pulvinar in non-human primates demonstrated neuronal activity reflecting the alternation between sensory sampling and motor planning phases (177,238,243) (Fig. 7). This rhythmic sampling is coordinated by oscillations in the theta-band modulating alpha oscillations which then impose inhibitory windows important for when and where attention is deployed. Collectively, this body of work integrates thalamic, cortical, and behavioural evidence into a rhythmic theory of attention that alternates between sampling and shifting states associated with respectively sensory processing and shifts in covert or overt spatial attention. In the sampling state, reduced alpha oscillations serve to route neural communication whereas in shifting state sensory processing is inhibited.

This collection of studies points to a strong role for alpha oscillations in the allocation of spatial attention in which they serve to suppress the processing of unattended visual inputs. While the alpha oscillations are under top-down control, it is debated to what extent they are controlled by the anticipation of distractors or rather the allocation of attention to targets and thereby indirect increase for unattended stimuli. Recent evidence points to the latter being the dominant effect (Jensen 2024; Bonnefond & Jensen 2025). Finally, this top-down control has been shown to be dynamic and possibly governed by intrinsic oscillatory mechanisms.

### 4.3 Modulation of alpha oscillations by auditory and somatosensory spatial attention

The aforementioned studies on alpha inhibition and spatial attention have been investigated on the visual domain. We here discuss findings generalising these mechanism other sensory modalities. Indeed, alpha oscillations have been investigated using somatosensory spatial attention tasks. Early work using MEG has reliably identified the 10 Hz component of the so-called rolandic mu-rhythm to the somatosensory cortex (244). As for the posterior alpha rhythm, the somatosensory ∼10 Hz rhythm is also linked to functional inhibition as it correlates negatively with the BOLD signal from sensorimotor regions (72,216) and neuronal firing (245). This sensorimotor rhythm is modulated by attention to either left- or right-hand tactile input in a manner very similar to the posterior alpha rhythm (193,246–251). While these oscillations are clearly under top-down control, it remains an open issue whether they are controlled by direct or indirect mechanisms, and which networks exercise the control. A study pointing to a direct control mechanism demonstrated that the somatosensory anticipatory alpha activity can increase to suppress distracting input (249).

Strong modulations have also been found in the alpha-band when auditory spatial attention was manipulated. When attention was allocated to auditory input from the left, alpha oscillations are decreased in right auditory cortical regions and vice versa (252,252–254). The neuronal activity associated with the allocation of auditory attention can however be difficult to isolate from supramodal effects as the modulation of posterior alpha oscillations in auditory tasks include posterior parietal regions. This was for instance the case in an EEG study investigating attention allocated to a left or right speech stream (255). Another MEG study reported a retinotopic distribution of parieto-occipital alpha oscillations in response to the allocation of auditory attention to a particular sound direction source arranged in a circular array around the participant (256). Beyond the supramodal effect there is evidence for alpha oscillations generated in auditory cortex using human intracranial recordings. Specifically a study using human intracranial recordings found that the magnitude of alpha oscillations in the auditory cortex increased when auditory stimuli were to be ignored (257). As such the allocation of spatial attention across modalities includes control mechanisms involving a supramodal system in parietal cortex that interacts with sensory-specific control systems (258).

### 4.4 Cross-modal interactions

The studies above make a strong case that the alpha oscillations reflect the allocation of computational resources in the visual, auditory and somatosensory domains. The top-down driven decrease of alpha oscillations in the hemisphere processing the attended stimuli and the relative increase in the other hemisphere suggest a push-pull mechanism between the hemispheres. However, could the alpha oscillations also reflect competitive interactions between sensory modalities? In 1944 Lord Adrian reported on an EEG study in which participants had to allocate attention to the speech input (75). Attending to speech, compared to visual input, resulted in an increase in power of posterior alpha oscillations. The alpha rhythm was interpreted to reflect a state of inattention which *“*…*fills those parts of the cortex which is for the moment unemployed.”* While these findings were based on a few observations, they were later reproduced in EEG studies using 64 electrodes and multiple participants (81). Again, a posterior alpha power increase was observed when attention was cued to auditory input. Similar findings were shown using MEG (259). Importantly, this study also demonstrated an increase in alpha power in the superior temporal gyrus when attention was allocated to the visual modality. Related findings have been reported when considering the somatosensory system. An MEG study on somatosensory working memory demonstrated that posterior alpha power increased during the maintenance of tactile stimuli (260). These studies suggest task-dependent competitive push-pull interactions between different sensory modalities. This collection of studies is compatible with the alpha inhibition hypothesis as the relative increase in alpha oscillations serves to suppress input from the unattended sensory modality.

Competitive interactions between the dorsal and ventral stream might also be reflected by alpha oscillations. In a working memory task, participants were asked to maintain either the identity or the orientation of a face presented for a few seconds. Maintaining the face identity supposedly engaged the ventral stream in the temporal cortex and resulted in an increase in alpha power over posterior parietal regions. However, maintaining the face orientation engaging the dorsal stream including the parietal cortex resulted in a relative alpha power decrease (261). Competitive interactions have also been found in terms of feature attention using EEG. Participants were cued to attend to either the colour or direction of motion of an upcoming moving-dot-kinematogram. Based on source modelling, it was demonstrated that alpha power increased in the dorsal stream when attending to colour relying on the ventral stream. Likewise, alpha power increased in the ventral stream, thus engaging the dorsal stream (262).

These findings suggest that alpha activity serves to resolve the competition across sensory modalities and between the dorsal versus ventral stream of the visual cortex; this is in general achieved by suppressing the unattended sensory modality or stream.

### 4.5 Working memory and distractor suppression

The cognitive studies mentioned so far make a strong case for a mechanistic role of alpha oscillations in allocating resources in attention tasks associated with sensory processing. One might also ask if the oscillations also reflect the allocation of internal resources, such as those required in working memory tasks where information must be maintained for shorter periods. Indeed, alpha oscillations are strongly modulated during working memory operations and synchrony betweem alpha sources is predicitve individual working memory capacity (158). This raises the question of whether the alpha oscillations mainly serve to allocate resources by inhibition, or if they also play an active role in the maintenance of working memory?

Several studies point to alpha oscillations playing an inhibitory role suppressing sensory information to prevent interference with working memory maintenance. As mentioned earlier, alpha oscillations have been found to remain strong during working memory maintenance (78) and they increase with working memory load (70,79,263) (Fig. 2). This resulted in the hypothesis that alpha oscillations serve to inhibit distracting information which was confirmed in several studies. When the distractors could be anticipated, alpha power increased just prior to the distractor onset. Furthermore, this increase predicted a behavioural reduction of distractor interference (82,83,264,265). The role of alpha oscillations for the allocation of resources was also confirmed using retro-cuing paradigms. In those studies, stimuli were presented in the left and right hemifield. A cue then prompted the retrieval of either the left or right stimuli previously presented. If the cue for instance prompted retrieval of an item from the left hemifield, this resulted in alpha decrease over the right hemisphere and a relative increase over the left hemisphere (266,267). The retro-cueing paradigms suggest that the alpha oscillations can partake in the internal selection of working memory representations.

Finally, it has been proposed that oscillations in the alpha-band serve to organise multiple working memory representations. This has been demonstrated using human intracranial recordings (268). In this study participants were asked to maintain three letters for a period of two seconds in a Sternberg task. The core finding was that some electrodes showed a selectivity increase in the gamma-band to specific letters, and this gamma-band activity was locked to the phase of 8 Hz alpha (or theta) oscillations. Importantly, there was an ordering with respect to the phase of the alpha oscillations: letters from early in the list had gamma burst early in the cycle, and letters from later in the list activated at later alpha phases. These findings suggest that beyond inhibiting sensory incoming information, the alpha oscillations might also serve to organise internal working memory presentations by a phase-coding scheme. It remains to be determined if the 8 Hz rhythm reflects slow alpha oscillations that might have slowed down due to the epilepsy or if they reflect a phenomenon different from the classical alpha rhythm. If the former, one could hypothesize that the alpha oscillation support the temporal organisation of intrinsic neuronal processing while at the same time serve preventing the in-flow of sensory input.

### 4.6 Long-term memory

As alpha oscillations have been shown to gate sensory information during spatial attention and working memory tasks, it might not be surprising that they also play a role for routing information in episodic long-term memory tasks. We will here highlight a few of studies on long-term memory underscoring how regional specific suppression by alpha oscillations can support encoding and recall. In an MEG study on long-term memory, it was demonstrated that when a cue indicating whether items should be encoded or not, stronger alpha oscillations were observed prior to items not to be encoded. These alpha oscillations further modulated the ongoing gamma oscillations associated with visual item processing (269). Another study demonstrated that posterior alpha power increased in the encoding interval after presentation during which items were rehearsed and incorporated into long-term memory. When long-term memory later was probed, the increase during encoding predicted the items later remembered (270). Beyond encoding, the alpha oscillations have also been shown to play a role for long-term memory recall. In an EEG study, object to be encoded in long-term memory were presented in the left or right hemifield (271). The objects were superimposed on a picture that later served as a cue. During the cued recall in a subsequent session, the alpha power decreased contralaterally to where the items were presented during encoding. This demonstrates that alpha oscillations can serve to allocate the computational resources of internal processes, such as the instantiation of memory representations. Beyond gating, alpha oscillations have been implicated in the neuronal processes directly associated with semantic memory processing. For instance, it has been demonstrated that upper alpha-band oscillations are depressed during semantic judgments (56). This effect was proposed to reflect thalamocortical interactions coordinating the retrieval of long-term memory representations (272).

In sum, these findings speak to neocortical alpha oscillations reflecting the encoding and recall of long-term memory representations. In particular, the decrease in alpha oscillations in the input stream might reflect memory encoding and recall whereas the increase reflects active rehearsal. The mechanisms at play in neocortical areas interact with the hippocampus involved in the actual synaptic encoding of the memory items (273).

### 4.7 Language and speech

The gating role of the alpha oscillations as detailed above has been centred around the routing of information in sensory or extra-sensory regions. The strong hemispheric separation of these regions in regard to visual hemifields has allowed for making a strong case for the functional role of the oscillations in attention and memory tasks with a spatial component. However, are alpha oscillations also important for the allocation of computational resources in neocortical areas associated with higher level cognition, for instance, speech comprehension and production? This question has been addressed directly or indirectly in several EEG and MEG studies. For instance, a large number of language tasks have been conducted in which sentences are presented visually word-by-word while the ongoing MEG or EEG data are recorded. In one study using a N400-type of paradigm, sentences were embedded with congruent or incongruent words. The presentation of incongruent compared to congruent words resulted in a decrease of alpha power over the left hemisphere (274). Other studies relied on sentences that were ending with target words that were either predictable or not from the sentence context. The key findings for both visual and auditory presentations, were that prior to predictable words, the alpha oscillations were relatively decreased in the left language network (275–277); but see (278). MEG studies revealed alpha oscillations that decreased in the left inferior frontal cortex, left posterior temporal region, and visual word form area (276). Importantly, the alpha power in the temporal and visual word form areas correlated negatively with left frontal gamma power for the sentences with a constraining context. This effect might reflect the initiation of an anticipatory unification process. These findings suggest that the modulation of alpha oscillations serves to allocate computational resources in the left inferotemporal and left posterior-temporal cortices during language tasks. In particular, alpha power decreases in the left hemisphere language network with increased task demands.

Extending these principles, other studies demonstrated that alpha-band (8–12 Hz) power tracks speech intelligibility in opposing, task-dependent ways. In a speech comprehension study orthogonally degrading single words in their temporal envelope and spectral detail showed that successful comprehension was predicted by late posterior alpha suppression (279); both the magnitude and topography of the decrease scaled with available acoustic detail. Source localisation implicated superior parietal, prefrontal, and anterior temporal regions, consistent with alpha suppression indexing enhanced sensory–perceptual processing when intelligibility is higher. By contrast, another study used an irrelevant-speech paradigm in which listeners maintained digit sequences while ignoring a concurrent degraded sentence (280). Here, alpha power increased when listeners’ goals required shielding working memory from distraction; critically, these modulations were driven by the attend-versus-ignore goal rather than acoustic degradation per se, marking alpha as a proxy for top-down attentional control. Taken together the modulation of the alpha activity depends on the listener’s goal: alpha suppression appears to facilitate extraction of acoustic detail and thus intelligibility, whereas alpha enhancement reflects the effort to inhibit task-irrelevant speech.

### 4.8 Alpha oscillations and clinical considerations

Over the last decades there have been numerous studies relating brain oscillations including alpha-band activity to both neurological and psychiatric disorders. As these studies have been extensively reviewed (281–284), we will here focus on disorders where an aberrant modulation of alpha oscillations have been identified in attention and working memory task. This is motivated by the fact that modulation of alpha-band activity is particularly robust in these tasks given the strong element of executive control.

**Figure 8:**
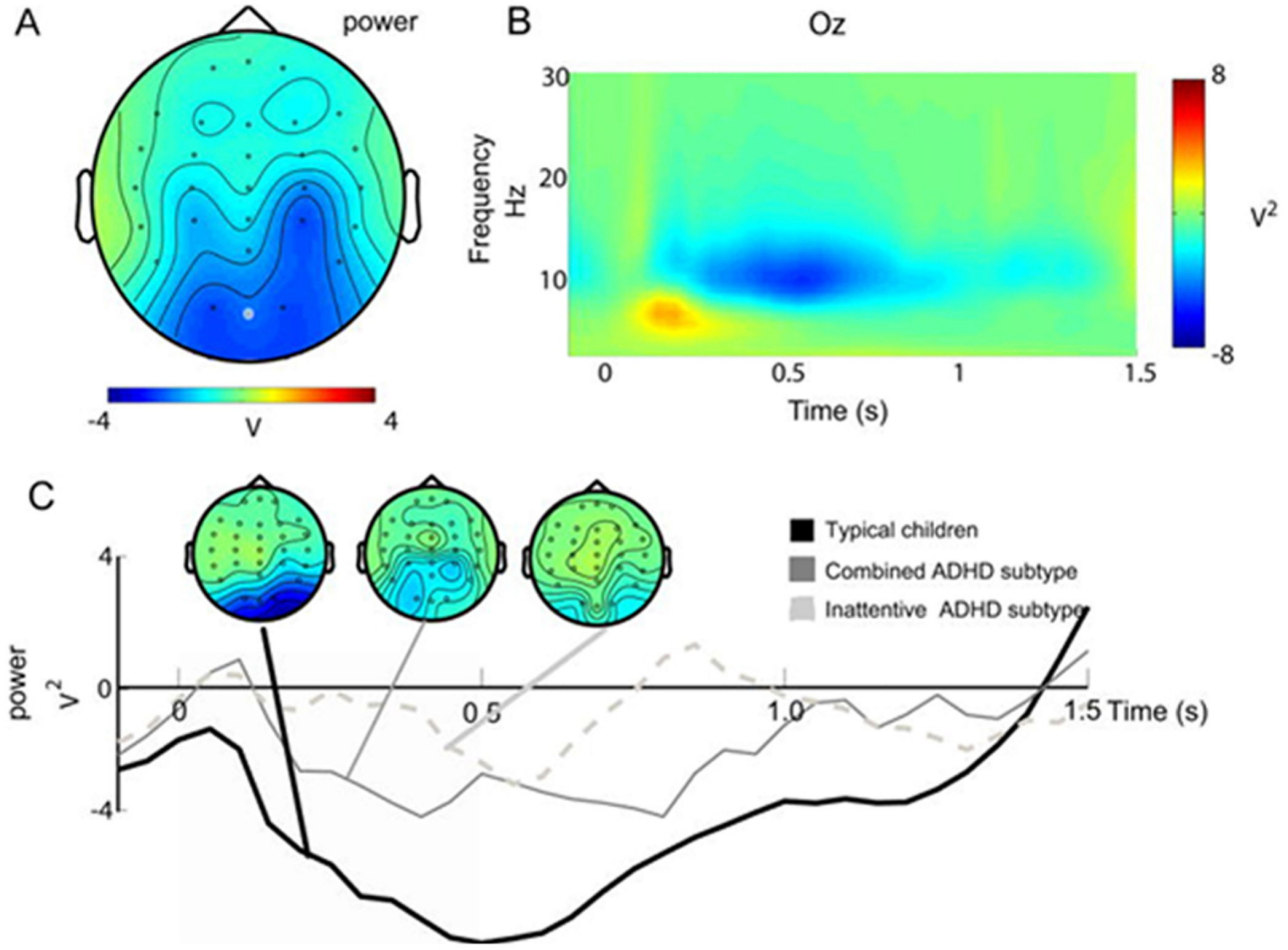
An EEG study showing differences in post-cue alpha suppression, a marker of visual cue processing, in individuals with ADHD and typically developing children. Adolescents with ADHD (especially the inattentive (IA) subtype) showed reduced occipital alpha suppression following response-preparation cues, reflecting weaker engagement of visual attentional mechanisms. A) The topography of post-cue alpha reduction across all groups. B) The cue-locked time-frequency spectra at occipital electrode Oz. C) The time course of alpha activity, with typically developing (TD) adolescents exhibited the strongest suppression in the 0–500 ms window, and IA adolescents the weakest. These oscillatory patterns align with broader findings that ADHD subtypes show distinct neural signatures, with IA displaying impaired visual cue processing and both subtypes showing altered top-down control. Reproduced from (285); used with permission.

Several cognitive problems and disorders are associated with problems in working memory and attention. One example being attention deficit and hyperactivity disorder (ADHD), which amongst others is associated with distractibility at the expense at keeping focus on a given task. One might therefore ask if ADHD is related to a decreased ability to modulate alpha oscillations in tasks requiring the allocation of spatial attention (286). This was tested in an MEG study in which adults diagnosed with ADHD were compared to a control group, and the participants were asked to perform a spatial attention task (287). The core finding was that the ADHD group had a decreased ability to sustain the hemispheric lateralisation of the alpha-band oscillations when being cued to the left, resulting in a behavioural bias to the right visual hemifield. A similar study was conducted in children diagnosed with ADHD. In this group, the boys had difficulties in modulating alpha oscillations in a spatial attention task (288). Related findings have been found in children diagnosed with ADHD engaging in working memory operations. Specifically, the ADHD group showed a reduced alpha power depression during working memory encoding compared to a control group (289). Importantly, the reduced alpha power depression in the ADHD group was subsequently found to predict ADHD symptoms and reduced reading comprehension and executive function (290). Another study employing a cross-modal paradigm found that cueing to either visual or auditory stimuli was associated with a frontal theta power increase and a modality-specific posterior alpha power decrease in healthy controls. These effects correlated over trials and were interpreted to reflect top-down attentional control; however, this effect was reduced in the children diagnosed with ADHD (291). An EEG study on adolescents with ADHD compared inattentive and combined subtypes. Using a paradigm with a cued flanker task, the inattentive subtype exhibited reduced occipital alpha suppression, suggesting weaker visual cue processing (Fig. 8), whereas the combined subtype shows diminished motor-related beta suppression, reflecting impaired motor preparation. Both groups display reduced frontal-posterior coupling in theta–alpha rhythms, highlighting deficits in top-down control (285). Together, these findings reveal subtype-specific neural signatures and support the use of oscillatory markers for refining ADHD characterization. In sum, as confirmed by a meta-analysis (292), children with ADHD have widespread alterations in the ability to modulate alpha oscillations during a range of neurocognitive tasks. These studies represent a few examples on how alpha oscillations can be used to investigate the neuronal mechanisms associated with neurodiversity as well as improve the characterisation of subtypes of conditions.

Ageing is associated with a decline in attention and working memory performance. Several studies have investigated whether these decreased abilities are reflected by changes in oscillations in the alpha-band. In an MEG study, elderly participants were engaged in a spatial working memory task in which they had to maintain visuo-spatial stimuli presented in both hemifields. They were probed to maintain the stimuli presented in either the left or right visual hemifield. The key finding was that the elderly compared to a younger control group had a reduced ability to modulate posterior alpha oscillations in regard to the spatial cue; however, this was explained by the alpha oscillations being decreased over both hemispheres (293). Given that a reduction in alpha oscillations is associated with increased neuronal engagement, these findings suggest that the elderly are compensating for diminished function by engaging posterior cortex but not in a spatially specific manner. An MEG study based on retro-cuing reported on a transient decrease in lateralized alpha power following a spatial cue (267). This modulation of alpha power was retained in older participants albeit working memory performance was reduced suggesting the performance issues in the elderly is not associated with flexible control *per se*. Other studies indicate that working memory deficits with ageing are associated with aberrant resting-state activity in the alpha and gamma -and. Specifically long-range coupling between frontal alpha oscillations and posterior >30 Hz activity was reduced with age (294). This reduction was predictive of age-related working memory decline.

There are numerous studies that have investigated changes in brain oscillations related to dementia both during rest and tasks. In general, the magnitude of posterior alpha oscillations is reduced with dementia whereas there is a widespread increase in activity in the delta and theta-band (284,295). It is debated to which extent this should be considered a slowing of the alpha oscillations, or the increase of specific sources in the theta-band. However, after reviewing the literature it was found that these changes were not sufficiently robust to be used for diagnosis albeit the changes might provide mechanistic insight into the neuronal mechanisms associated with dementia (296). Possibly future work in which the modulation of alpha-band oscillations is related to dementia in the context of attention and working memory tasks would prove more diagnostically informative.

We have here highlighted a few studies demonstrating how the mechanistic role of alpha oscillations can be investigated in individuals with problems associated with neurodiversity and age-related cognitive decline. Evidently the literature in general is vast on relating alpha oscillations to disorders. While part of this literature is somewhat descriptive, the studies that do provide mechanistic insight are typically consistent with the notion that problems in inhibiting task-irrelevant brain structures can result in reduced behavioural performance (283,286).

## 5 Emerging themes

We have reviewed the functional role of alpha oscillations and the physiological mechanisms that support them. Oscillations in the alpha-band are dominant both during resting and when the brain is engaged. The emerging consensus is that these oscillations serve to suppress brain regions that are not involved in a particular task. This suppression serves to allocate computational resources to brain areas that are active and supporting the task at hand. Consequently, alpha oscillations function at the network level in a task-dependent manner. In this section, we will discuss emerging themes based on this general framework to further explore the role of brain oscillations.

### 5.1 Travelling waves

The neocortex is composed of a dense network of excitatory and inhibitory neurons with both local and long-ranging connections. The local excitation can activate neighbouring neurons and thereby result in travelling waves (297,297–299). Given the wide-spread presence of alpha oscillations, the travelling waves may be reflected by oscillations with a systematic change in phase across cortex. Indeed there are several reports of travelling waves in the alpha-band observed in humans dating back to EEG recordings in the 70s (297). Using ECoG recordings, neocortical alpha waves were observed in posterior brain regions during rest (182). High-frequency gamma-band activity was locked to the phase of the alpha oscillations and thereby travelled with the waves. Further studies based on EcoG recordings demonstrated that the propagation was task-dependent and more consistent when a given task was performed well (300). Using ECoG recordings in marmoset monkeys, 8 Hz travelling waves elicited by saccades travelled anterior-posteriorly in the dorsal stream and then posterior-anteriorly in the ventral stream (301). Task-dependent modulations of alpha-band oscillations have also been reported in human EEG studies in which the direction of travel of the alpha oscillations was investigated (302,303). During operations requiring top-down control, the alpha waves propagated from front to back; however, during sensory processing they mainly propagated from back to front (303). From a mechanistic perspective, alpha waves have been related to a model on predictive coding involving top-down predictions and feed-forward of prediction errors. The timing of this processing required an integration between prediction and sensory input and was proposed to be controlled by travelling waves in the alpha-band (304)

While travelling waves in the alpha-band have indeed been identified in human ECoG recordings, there is a debate on how reliable they can be identified in EEG and MEG data. The core problem is that EEG and MEG scalp recordings result from a linear mixing of the neuronal sources due to volume conduction and field spread. As such, two sources in different parts of the brain oscillating out of phase at 10 Hz, can generate a travelling wave at the scalp level (305). This phenomenon results in ambiguities when interpreting EEG and MEG data in terms of travelling waves. There are several projects in development to uncover when it is possible to identify spurious from physiological travelling waves (306).

Finally, more complex spatio-temporal dynamics anchored in the propagation of neuronal excitability have been proposed to support large-scale brain computations. This research line is based on computational modelling and experimental work considering both short- and long-range coupling suggesting that the brain network can exhibit dynamics with vortices and turbulence (307,307–310). In future work, it would be interesting to explore the interaction between travelling alpha waves at different spatial scales and the emergence of turbulent dynamics.

### 5.2 Alpha oscillations and saccades

Since alpha oscillations are modulated in visual tasks and most strongly observed over posterior brain regions, they have typically been associated with visual processing. Given that alpha oscillations modulate neuronal activity in a phasic manner, this poses an interesting conundrum: given how fast the visual system operates, how come visual processing is ‘clocked’ by a relatively slow 10 Hz alpha rhythm? This question should be considered given that visual saccades occur 3 to 4 times per second in natural settings. This leaves about 3 to 4 alpha cycles between each saccade. These timing issues could be resolved by saccade onsets being locked to the phase of the ongoing 10 Hz rhythm thus supporting a mechanism in which visual input arrives at the excitatory phase of the ongoing alpha oscillations.

**Figure 9:**
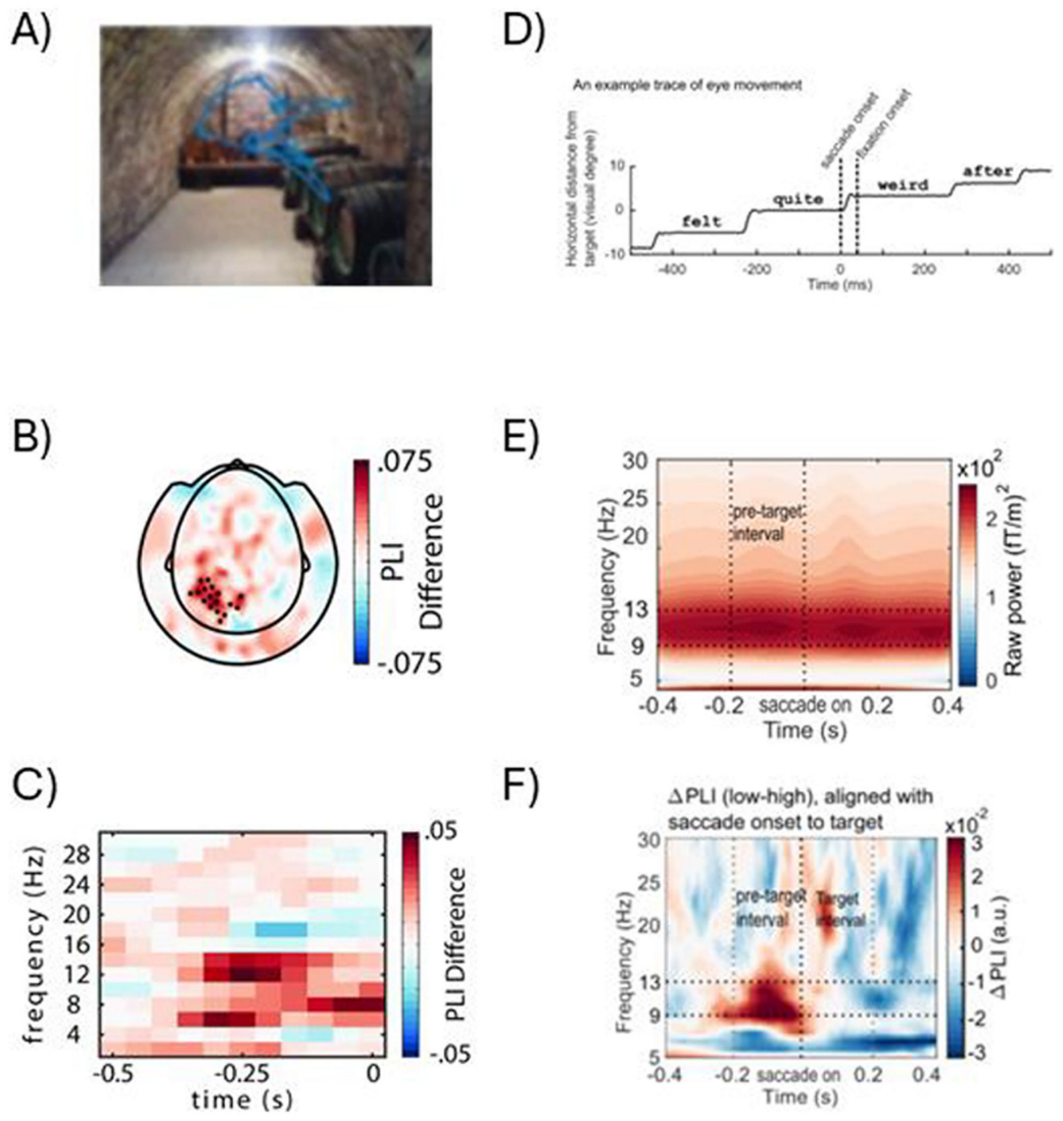
Human findings demonstrating that saccade onsets are locked to the phase of ongoing alpha oscillations. A) MEG and eye-tracking data were recording while participants explored images to be remember. The MEG epochs were aligned to saccades onset and then analysed. B) Significant phase-locked in the 12-14 Hz alpha-band was observed over posterior sensors. C) A time-frequency representation of the phase-locking index over epochs locked to saccade onsets. The phase-locking was constrained to the 12-14 Hz band about 200 ms prior to saccade onset and it was stronger for images later remembered compared to forgotten. D) MEG and eye-tracking data were recorded in a natural reading paradigm. E) A time-frequency representation of power averaged over epochs locked to saccade onset. The alpha power remained strong during reading, i.e. it was not depressed or blocked with saccades onset. F) Robust phase-locking was observed in the alpha-band for epochs aligned to saccade onset. The phase-locking was stronger for saccade prepared toward low- compared to high-frequency words in the sentence. Reproduced from (312,313)

Indeed, there is empirical work in humans and non-human primates linking eye movements to alpha oscillations. This work goes back to the findings of Olof Lippold arguing that alpha oscillations might be a consequence of eye-muscle tremors (21). While this notion has been disproven, Lippold did conduct a set of studies linking eye movements to alpha oscillations. For instance, he demonstrated that mechanical movements of the eyeball resulted in alpha oscillations being elicited (21). Furthermore, cooling of the p orbit would impact the alpha oscillations (20). Later work in humans relying on intracranial EEG recordings and MEG has investigated the link between the phase of alpha oscillations and saccades in visual exploration and reading tasks (311–313). These studies have demonstrated that saccades onsets indeed are more likely to occur at certain phases of the alpha oscillations (Fig. 9). In a memory task where visual scenes were explored, this coupling predicted to which extent the image was encoded in memory (312). A reading study revealed that the coupling was stronger when participants were preparing saccades towards low-frequency words (313). These findings demonstrate that saccades are locked to the phase of alpha oscillations possibly allowing for post-saccadic visual information to arrive at the excitatory phase of the oscillations to support visual perception. There is also complementary work in non-human primates suggesting that alpha oscillations in V4 might link current and upcoming receptive field (314). In sum, these findings suggest that alpha oscillations serve to coordinate visual and eye-movements.

Other work based on EEG has linked microsaccades to alpha oscillations (315). Microsaccades are small eye movements often modulated by the direction of covert attention. This raises the question of whether there is a relationship between microsaccades and alpha-band modulations. In a recent EEG study, participants memorised items presented in the left and right hemifields while the eye movements were recorded under strict fixation. Upon cuing, posterior alpha exhibited hemispheric lateralisation even in trials without any microsaccades. When microsaccades did occur, their direction and timing covaried with alpha modulation. Overall, microsaccades are functionally related but not obligatory for human neural attention effects (316). A follow up study showed that transient lateralisation of posterior alpha power is aligned to microsaccades: alpha increases ipsilateral to the movement direction and decreases contralateral, peaking within ∼250 ms after onset. This effect appears for both “start” microsaccades (away from fixation) and “return” microsaccades (back to fixation) and is driven primarily by an ipsilateral alpha increase; the response also has a phase-locked (ITPC) component reflecting an alpha phase reset after fixation (317). During internal refocusing of attention, alpha lateralisation and a bias in (micro)saccade direction each scale with cue reliability, and the oculomotor dynamics is predominantly driven by fixational microsaccades — reinforcing that alpha and microsaccades provide tandem readouts of internal spatial selection (318). Extending beyond discrete trial epochs, simultaneous MEG and eye tracking reveal a broader cortico-ocular coupling: gaze variability co-varies with alpha/beta (10–20 Hz) power decreases in visual cortex and predicts subsequent memory. This co-variation holds both during encoding and prestimulus baselines, suggesting a domain-general linkage between ocular sampling and oscillatory cortical excitability that supports mnemonic outcomes (319). Together, these studies converge on an account in which alpha oscillations help coordinate the temporal alignment of visual-cortical excitability with oculomotor sampling. Microsaccades can shape the alpha dynamics, yet robust alpha lateralisation of spatial attention can arise without eye-movements, consistent with partially shared—but not obligatory—oculomotor control of attentional selection.

Evidently in real-life situations, saccades and visual processing are tightly coordinated. Portable EEG as well as MEG based on Optically Pumped Magnetometers (320) are resilient to head movements, allowing brain recordings in ecologically realistic settings. These tools can be combined with cameras allowing to detect eye and limb movements thus investigating the role of alpha oscillations in relation to motor control in more natural settings.

In conclusion, recent work suggests a strong connection between the phase of alpha oscillations and the timing of motor operations. This is particularly evident for the control of eye-movements as supported by studies on saccades and microsaccades. The control could be implemented by alpha oscillations coordinating the interaction between sensory and motor regions by pulsed inhibition being simultaneously released across the involved regions. The proposed framework prompts a rethinking of the functional role of alpha oscillations from primarily controlling sensory flow to coordinating visual processing and eye-movements. In future work it would be of great interest to uncover the extended control network supporting this coordination. Key regions in this network include the frontal eye field and the superior colliculus.

### 5.3 Development and genetics

**Figure 10:**
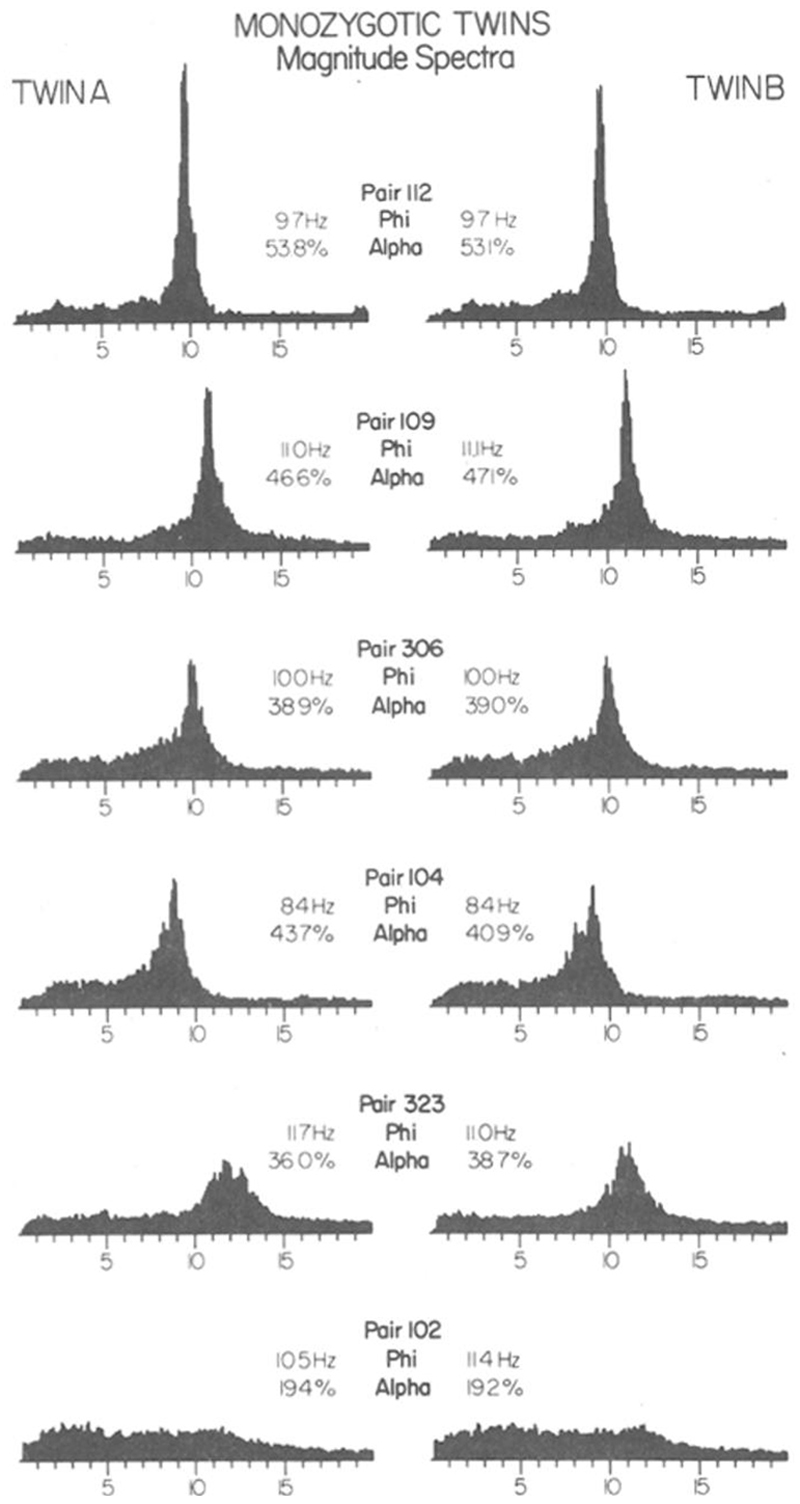
The spectral properties and peak frequency of the alpha oscillations are remarkably similar across monozygotic twins. This demonstrates a high degree of heritability thus motivating investigation into the genetic basis of the rhythm generation. Reproduced from (337); used with permission.

Investigations of alpha oscillations across the lifespan have shown systematic changes in the frequency (321–323). In infants and toddlers, the posterior oscillations are about 6-9 Hz (324). They then accelerate with age and mature to about 8-12 Hz in the early teenage years (325). The changes in frequency have been related to cognitive maturity (326,327); for instance, it has been shown that a higher alpha frequency is associated with precocious reading abilities (328). At older ages, the alpha frequency starts to decrease systematically and is related to cognitive performance (322,329). As we previously pointed out, the frequency of the alpha oscillations is intimately linked to the time constants associated with the underlying physiology. In particular, the kinetics of the synaptic and membrane receptors involved in generating the alpha frequency determine the specific frequency; however, white matter connectivity reflecting myelination might also play a role (322,330). As such, it would be of great interest to uncover the changes in neurophysiology over the lifespan that determine the change in alpha frequency. This might be particularly important for assessing early development in order to provide reliable markers of cognitive maturity. New technical developments based on MEG using Optically Pumped Magnetometers (OPMs) hold a strong promise for improving recordings in infants and children (331–334). This is because sensor arrays using OPMs can be adapted to individual head sizes being particularly advantageous for paediatric recordings. Beyond providing mechanistic insight on brain development such insight might help to guide interventions. Finally, a topic that deserves further attention is the genetic factors impacting the alpha rhythm. As shown in Fig. 10, the peak frequency of the alpha rhythm is remarkably similar between monozygotic twins showing a high degree of heritability (335–341).

The high degree of heritability suggests that specific genes are involved in determining the spectral properties of the alpha oscillations. It would be of great interest to identify the chromosomal location of the genes determining spectral properties to uncover the associated proteins. This will provide pointers to the physiological mechanism responsible for generating the alpha oscillations. Such research could be done by linkage analysis within families in combination with genotyping and EEG/MEG recordings. Given the mechanistic role of alpha oscillations for perception, behaviour and clinical symptoms, this research line holds the promise of bringing genetics and physiology to provide mechanistic insight into cognition and disorders.

## 6 Conclusion

We have reviewed the functional role of alpha oscillations in the context of cognition and the associated physiological mechanisms. While alpha oscillations were initially thought to be related solely to idling and rest, it is now evident that they play a crucial role in the active brain. Numerous studies have shown that alpha oscillations reflect functional inhibition and are essential for allocating resources during cognitive processing. This allocation is achieved by task-dependent engagement or disengagement of specific brain regions. The inhibition by the alpha oscillations occurs in a pulsed manner, thereby providing a phasic modulation of the neuronal activity. The allocation of resources reflected by region-specific modulations in the alpha-band has been observed in almost all tasks investigated spanning from simple perception to speech comprehension. As confirmed by both human MEG studies and intracranial animal investigations, the alpha oscillations are observed in a wide range of regions, thus serving a widespread role in allocating resources and filtering by inhibition. Despite the insights gained regarding alpha oscillations, further research is needed, particularly to understand the control mechanisms of these oscillations and the underlying physiological processes. Emerging themes include a deeper comprehension of how alpha oscillations develop in children and how they relate to cognitive maturity. Moreover, we need to better understand how alpha oscillations function at the network level including the interactions between different brain regions, and the role of travelling waves. Recent research specifically highlights the close coupling between saccades and alpha oscillations in the context of visual processing. This insight opens the possibility of investigating the role of alpha oscillations in more ecologically valid tasks such as natural visual processing and reading.

## Clinical highlights

The role of alpha oscillations has been investigated in individuals with various disorders. This research has led to the understanding that attention problems are reflected in an impaired ability to modulate alpha oscillations during spatial attention and working memory tasks. Consequently, studying alpha oscillations holds the promise of providing insights into the network-level mechanisms associated with cognitive problems. Moreover, the properties of alpha oscillations are strongly hereditary, opening the door for future investigations that could causally link the genetic basis of neuronal dynamics to neurological and psychiatric disorders. Because alpha oscillations change markedly during early development—and many disorders emerge in infancy and childhood—uncovering age-related trajectories may reveal mechanisms and inform early intervention.

## References

[1] Berger H (1929). Über das Elektrenkephalogramm des Menschen. Archiv f Psychiatrie.

[2] Millett D (2001). Hans Berger: From Psychic Energy to the EEG. Perspectives in Biology and Medicine.

[3] Meynert T (1874). Zur Mechanik des Gehirnbaues. Braumüller.

[4] Lehmann A (1892). Die Hauptgesetze des menschlichen Gefühlslebens: eine experimentelle und analytische Untersuchung über die Natur und das Auftreten der Gefühlszustände nebst einem Beitrage zu deren Systematik. Reisland.

[5] Berger H (1901). Zur Lehre von der Blutzirkulation in der Schädelhöhle des Menschen namentlich unter dem Einfluss von Medikamenten: (Experimentelle Untersuchungen) … G. Fischer.

[6] Berger H (1910). Untersuchungen über die Temperatur des Gehirns.

[7] Mosso A (1894). Die Temperatur des Gehirns: Untersuchungen. Veit.

[8] Stone JL, Hughes JR (2013). Early History of Electroencephalography and Establishment of the American Clinical Neurophysiology Society. Journal of Clinical Neurophysiology.

[9] Adrian ED, Matthews BH (1934). The Berger Rhythm: Potential Changes From the Occipital Lobes in Man. Brain.

[10] Ginzberg R (1949). Three Years with Hans Berger A Contribution to His Biography. Journal of the History of Medicine and Allied Sciences.

[11] Tönnies JF (1933). Die Ableitung bioelektrischer Effekte vom uneröffneten Schädel. Physikalische Behandlung des Problems. J Psychol Neurol.

[12] O’Leary JL (1970). Science.

[13] Aserinsky E, Kleitman N (1953). Regularly occurring periods of eye motility, and concomitant phenomena, during sleep. Science.

[14] Raichle ME (1998). Behind the scenes of functional brain imaging: A historical and physiological perspective. Proceedings of the National Academy of Sciences.

[15] Raichle ME (2009). A brief history of human brain mapping. Trends in Neurosciences.

[16] Adrian ED, Yamagiwa K (1935). The origin of the Berger rhythm. Brain: A Journal of Neurology.

[17] Mosher JC, Leahy RM, Lewis PS (1999). EEG and MEG: forward solutions for inverse methods. IEEE Trans Biomed Eng.

[18] Lippold O (1970). Origin of the Alpha Rhythm. Nature.

[19] Lippold OC, Novotny GE (1970). Is alpha rhythm an artifact?. Lancet.

[20] Lippold OC, Novotny GE (1968). Tremor of the extra-ocular muscles as the generator of alpha rhythm: cooling the orbit in man. J Physiol.

[21] Lippold OCJ (1973). The origin of the alpha rhythm.

[22] Enger PS (1957). The electroencephalogram of the codfish (Gadus callarias); spontaneous electrical activity and reaction to photic and acoustic stimulation. Acta Physiol Scand.

[23] Schadé JP, Weiler IJ (1959). Electroencephalographic Patterns of the Goldfish (Carassius Auratus L.)*. Journal of Experimental Biology.

[24] Segura ET, de Juan A (1966). Electroencephalographic studies in toads. Electroencephalography and Clinical Neurophysiology.

[25] Sugihara K, Gotoh J (1973). Depth-Electroencephalograms of Chickens in Wakefulness and Sleep. The Japanese Journal of Physiology.

[26] van Leeuwen WS, Kamp A (1969). Radio telemetry of EEG and other biological variables in man and dog. Proc R Soc Med.

[27] Lanoir J, Cordeau JP (1970). Spontaneous and rhythms spindles of the cat primary visual area during different stages of wakefulness and sleep. J Physiol (Paris).

[28] da Silva FH, van Lierop TH, Schrijer CF, van Leeuwen WS (1973). Organization of thalamic and cortical alpha rhythms: spectra and coherences. Electroencephalogr Clin Neurophysiol.

[29] Rougeul A, Corvisier J, Letalle A (1974). Electrocortical rhythms characteristic of the onset of natural sleep in the cat. Their relationship to motor activity. Electroencephalogr Clin Neurophysiol.

[30] Schürmann M, Demiralp T, Başar E, Başar Eroglu C (2000). Electroencephalogram alpha (8-15 Hz) responses to visual stimuli in cat cortex, thalamus, and hippocampus: a distributed alpha network?. Neurosci Lett.

[31] Li Y, Yu C, Zhou ZC, Stitt I, Sellers KK, Gilmore JH (2017). Early Development of Network Oscillations in the Ferret Visual Cortex. Sci Rep.

[32] Andersen P, Andersson SA (1968). Physiological Basis of the Alpha Rhythm. Appleton-Century-Crofts.

[33] da Silva FHL, van Lierop THMT, Schrijer CF, van Leeuwen WS (1973). Essential differences between alpha rhythms and barbiturate spindles: Spectra and thalamo-cortical coherences. Electroencephalography and Clinical Neurophysiology.

[34] Bollimunta A, Chen Y, Schroeder CE, Ding M (2008). Neuronal Mechanisms of Cortical Alpha Oscillations in Awake-Behaving Macaques. J Neurosci.

[35] Buffalo EA, Fries P, Landman R, Buschman TJ, Desimone R (2011). Laminar differences in gamma and alpha coherence in the ventral stream. Proceedings of the National Academy of Sciences of the United States of America.

[36] Spaak E, Bonnefond M, Maier A, Leopold DA, Jensen O (2012). Layer-Specific Entrainment of Gamma-Band Neural Activity by the Alpha Rhythm in Monkey Visual Cortex. Current Biology.

[37] van Kerkoerle T, Self MW, Roelfsema PR (2017). Layer-specificity in the effects of attention and working memory on activity in primary visual cortex. Nat Commun.

[38] Haegens S, Barczak A, Musacchia G, Lipton ML, Mehta AD, Lakatos P (2015). Laminar Profile and Physiology of the alpha Rhythm in Primary Visual, Auditory, and Somatosensory Regions of Neocortex. The Journal of neuroscience : the official journal of the Society for Neuroscience.

[39] Arroyo S, Lesser RP, Gordon B, Uematsu S, Jackson D, Webber R (1993). Functional significance of the mu rhythm of human cortex: an electrophysiologic study with subdural electrodes. Electroencephalogr Clin Neurophysiol.

[40] Toro C, Deuschl G, Thatcher R, Sato S, Kufta C, Hallett M (1994). Event-related desynchronization and movement-related cortical potentials on the ECoG and EEG. Electroencephalogr Clin Neurophysiol.

[41] Crone NE, Miglioretti DL, Gordon B, Sieracki JM, Wilson MT, Uematsu S (1998). Functional mapping of human sensorimotor cortex with electrocorticographic spectral analysis. I. Alpha and beta event-related desynchronization. Brain.

[42] Cooper R, Mundy-Castle AC (1960). Spatial and temporal characteristics of the alpha rhythm: a toposcopic analysis. Electroencephalogr Clin Neurophysiol.

[43] Lehmann D (1971). Multichannel topography of human alpha EEG fields. Electroencephalography and Clinical Neurophysiology.

[44] Chatrian GE, Petersen MC, Lazarte JA (1959). The blocking of the rolandic wicket rhythm and some central changes related to movement. Electroencephalogr Clin Neurophysiol.

[45] Jasper HH, Andrews HL (1938). Electro-Encephalography: III. Normal Differentiation Of Occipital And Precentral Regions in Man. Archives of Neurology & Psychiatry.

[46] Pfurtscheller G, Haring G (1972). The use of an EEG autoregressive model for the time-saving calculation of spectral power density distributions with a digital computer. Electroencephalogr Clin Neurophysiol.

[47] Pfurtscheller G, Aranibar A (1977). Event-related cortical desynchronization detected by power measurements of scalp EEG. Electroencephalogr Clin Neurophysiol.

[48] Pfurtscheller G, Lopes da Silva FH (1999). Event-related EEG/MEG synchronization and desynchronization: basic principles. Clinical Neurophysiology.

[49] Pfurtscheller G, Stancák A, Neuper C (1996). Event-related synchronization (ERS) in the alpha band--an electrophysiological correlate of cortical idling: a review. Int J Psychophysiol.

[50] Pfurtscheller G, Neuper C, Andrew C, Edlinger G (1997). Foot and hand area mu rhythms. Int J Psychophysiol.

[51] Neuper C, Wörtz M, Pfurtscheller G (2006). ERD/ERS patterns reflecting sensorimotor activation and deactivation. Prog Brain Res.

[52] Rihs TA, Michel CM, Thut G (2007). Mechanisms of selective inhibition in visual spatial attention are indexed by alpha-band EEG synchronization. Eur J Neurosci.

[53] Bahramisharif A, van Gerven M, Heskes T, Jensen O (2010). Covert attention allows for continuous control of brain-computer interfaces. Eur J Neurosci.

[54] Harvey BM, Vansteensel MJ, Ferrier CH, Petridou N, Zuiderbaan W, Aarnoutse EJ (2013). Frequency specific spatial interactions in human electrocorticography: V1 alpha oscillations reflect surround suppression. Neuroimage.

[55] Popov T, Gips B, Kastner S, Jensen O (2019). Spatial specificity of alpha oscillations in the human visual system. Hum Brain Mapp.

[56] Klimesch W, Schimke H, Schwaiger J (1994). Episodic and semantic memory: an analysis in the EEG theta and alpha band. Electroencephalogr Clin Neurophysiol.

[57] Klimesch W, Doppelmayr M, Pachinger T, Russegger H (1997). Event-related desynchronization in the alpha band and the processing of semantic information. Brain Res Cogn Brain Res.

[58] Sauseng P, Klimesch W, Stadler W, Schabus M, Doppelmayr M, Hanslmayr S (2005). A shift of visual spatial attention is selectively associated with human EEG alpha activity. Eur J Neurosci.

[59] Klimesch W (1996). Memory processes, brain oscillations and EEG synchronization. Int J Psychophysiol.

[60] Klimesch W (2012). α-band oscillations, attention, and controlled access to stored information. Trends Cogn Sci.

[61] Bruns A (2004). Fourier-, Hilbert- and wavelet-based signal analysis: are they really different approaches?. Journal of Neuroscience Methods.

[62] Le Van Quyen M, Foucher J, Lachaux J, Rodriguez E, Lutz A, Martinerie J (2001). Comparison of Hilbert transform and wavelet methods for the analysis of neuronal synchrony. J Neurosci Methods.

[63] Tallon-Baudry C (1999). Oscillatory gamma activity in humans and its role in object representation. Trends in Cognitive Sciences.

[64] Gevins A, Smith ME, McEvoy L, Yu D (1997). High-resolution EEG mapping of cortical activation related to working memory: effects of task difficulty, type of processing, and practice. Cereb Cortex.

[65] Hämäläinen M, Hari R, Ilmoniemi RJ, Knuutila J, Lounasmaa OV (1993). Magnetoencephalography---theory, instrumentation, and applications to noninvasive studies of the working human brain. Rev Mod Phys.

[66] Baillet S (2017). Magnetoencephalography for brain electrophysiology and imaging. Nat Neurosci.

[67] Hari R, Salmelin R, Mäkelä JP, Salenius S, Helle M (1997). Magnetoencephalographic cortical rhythms. Int J Psychophysiol.

[68] Dolan RJ (2008). Neuroimaging of cognition: past, present, and future. Neuron.

[69] Ritter P, Moosmann M, Villringer A (2009). Rolandic alpha and beta EEG rhythms’ strengths are inversely related to fMRI-BOLD signal in primary somatosensory and motor cortex. Human Brain Mapping.

[70] Scheeringa R, Petersson KM, Oostenveld R, Norris DG, Hagoort P, Bastiaansen MCM (2009). Trial-by-trial coupling between EEG and BOLD identifies networks related to alpha and theta EEG power increases during working memory maintenance. Neuroimage.

[71] Zumer JM, Scheeringa R, Schoffelen JM, Norris DG, Jensen O (2014). Occipital alpha activity during stimulus processing gates the information flow to object-selective cortex. PLoS Biol.

[72] Wilson R, Mullinger KJ, Francis ST, Mayhew SD (2019). The relationship between negative BOLD responses and ERS and ERD of alpha/beta oscillations in visual and motor cortex. Neuroimage.

[73] Drever J (1955). Some observations on the occipital alpha rhythm. Quarterly Journal of Experimental Psychology.

[74] Mundy-Castle AC (1957). The electroencephalogram and mental activity. Electroencephalography and Clinical Neurophysiology.

[75] Adrian ED (1944). Brain Rhythms*. Nature.

[76] Ray WJ, Cole HW (1985). EEG alpha activity reflects attentional demands, and beta activity reflects emotional and cognitive processes. Science.

[77] Krause CM, Lang AH, Laine M, Kuusisto M, Pörn B (1996). Event-related EEG desynchronization and synchronization during an auditory memory task. Electroencephalogr Clin Neurophysiol.

[78] Klimesch W, Doppelmayr M, Schwaiger J, Auinger P, Winkler T (1999). ‘Paradoxical’ alpha synchronization in a memory task. Brain Res Cogn Brain Res.

[79] Jensen O, Gelfand J, Kounios J, Lisman JE (2002). Oscillations in the alpha band (9-12 Hz) increase with memory load during retention in a short-term memory task. Cereb Cortex.

[80] Foxe JJ, Simpson GV, Ahlfors SP (1998). Parieto-occipital approximately 10 Hz activity reflects anticipatory state of visual attention mechanisms. Neuroreport.

[81] Fu KM, Foxe JJ, Murray MM, Higgins BA, Javitt DC, Schroeder CE (2001). Attention-dependent suppression of distracter visual input can be cross-modally cued as indexed by anticipatory parieto-occipital alpha-band oscillations. Brain Res Cogn Brain Res.

[82] Bonnefond M, Jensen O (2012). Alpha oscillations serve to protect working memory maintenance against anticipated distracters. Curr Biol.

[83] Payne L, Guillory S, Sekuler R (2013). Attention-modulated alpha-band oscillations protect against intrusion of irrelevant information. J Cogn Neurosci.

[84] Jensen O, Mazaheri A (2010). Shaping functional architecture by oscillatory alpha activity: gating by inhibition. Front Hum Neurosci.

[85] Klimesch W, Sauseng P, Hanslmayr S (2007). EEG alpha oscillations: the inhibition-timing hypothesis. Brain Res Rev.

[86] Mazaheri A, Jensen O (2010). Rhythmic pulsing: linking ongoing brain activity with evoked responses. Front Hum Neurosci.

[87] VanRullen R (2016). Perceptual Cycles. Trends Cogn Sci.

[88] Saalmann YB, Kastner S (2011). Cognitive and Perceptual Functions of the Visual Thalamus. Neuron.

[89] Gücer G, Niedermeyer E, Long DM (1978). Thalamic EEG recordings in patients with chronic pain. J Neurol.

[90] Lopes da Silva FH, Vos JE, Mooibroek J, Van Rotterdam A (1980). Relative contributions of intracortical and thalamo-cortical processes in the generation of alpha rhythms, revealed by partial coherence analysis. Electroencephalogr Clin Neurophysiol.

[91] Rougeul-Buser A, Buser P (1997). Rhythms in the alpha band in cats and their behavioural correlates. International Journal of Psychophysiology.

[92] Lorincz ML, Kekesi KA, Juhasz G, Crunelli V, Hughes SW (2009). Temporal framing of thalamic relay-mode firing by phasic inhibition during the alpha rhythm. Neuron.

[93] Lukashevich IP, Sazonova OB (1996). The effect of lesions of different parts of the optic thalamus on the nature of the bioelectrical activity of the human brain. Zh Vyssh Nerv Deiat Im I P Pavlova.

[94] Halassa MM, Kastner S (2017). Thalamic functions in distributed cognitive control. Nat Neurosci.

[95] Hughes SW, Lorincz M, Cope DW, Blethyn KL, Kekesi KA, Parri HR (2004). Synchronized oscillations at alpha and theta frequencies in the lateral geniculate nucleus. Neuron.

[96] Suffczynski P, Kalitzin S, Pfurtscheller G, Lopes da Silva FH (2001). Computational model of thalamo-cortical networks: dynamical control of alpha rhythms in relation to focal attention. Int J Psychophysiol.

[97] Huguenard JR (1996). Low-Threshold Calcium Currents in Central Nervous System Neurons. Annual Review of Physiology.

[98] Zhou Q, Godwin DW, O’Malley DM, Adams PR (1997). Visualization of Calcium Influx Through Channels That Shape the Burst and Tonic Firing Modes of Thalamic Relay Cells. Journal of Neurophysiology.

[99] Hughes SW, Crunelli V (2005). Thalamic mechanisms of EEG alpha rhythms and their pathological implications. Neuroscientist.

[100] Lorincz ML, Crunelli V, Hughes SW (2008). Cellular dynamics of cholinergically induced alpha (8-13 Hz) rhythms in sensory thalamic nuclei in vitro. The Journal of neuroscience : the official journal of the Society for Neuroscience.

[101] Hughes SW, Lőrincz ML, Blethyn K, Kékesi KA, Juhász G, Turmaine M (2011). Thalamic Gap Junctions Control Local Neuronal Synchrony and Influence Macroscopic Oscillation Amplitude during EEG Alpha Rhythms. Front Psychol.

[102] Lopes da Silva FH, van Lierop THMT, Schrijer CF, Storm van Leeuwen W (1973). Organization of thalamic and cortical alpha rhythms: Spectra and coherences. Electroencephalography and Clinical Neurophysiology.

[103] Chatila M, Milleret C, Rougeul A, Buser P (1993). Alpha rhythm in the cat thalamus. C R Acad Sci III.

[104] Saalmann YB, Pinsk MA, Wang L, Li X, Kastner S (2012). The Pulvinar Regulates Information Transmission Between Cortical Areas Based on Attention Demands. Science.

[105] Roux F, Wibral M, Singer W, Aru J, Uhlhaas PJ (2013). The phase of thalamic alpha activity modulates cortical gamma-band activity: evidence from resting-state MEG recordings. The Journal of neuroscience : the official journal of the Society for Neuroscience.

[106] Minami S, Oishi H, Takemura H, Amano K (2020). Inter-individual Differences in Occipital Alpha Oscillations Correlate with White Matter Tissue Properties of the Optic Radiation. eNeuro.

[107] Bastos AM, Briggs F, Alitto HJ, Mangun GR, Usrey WM (2014). Simultaneous recordings from the primary visual cortex and lateral geniculate nucleus reveal rhythmic interactions and a cortical source for gamma-band oscillations. The Journal of neuroscience : the official journal of the Society for Neuroscience.

[108] Bastos AM, Vezoli J, Bosman CA, Schoffelen JM, Oostenveld R, Dowdall JR (2015). Visual areas exert feedforward and feedback influences through distinct frequency channels. Neuron.

[109] Hoffman SJ, Dotson NM, Lima V, Gray CM (2024). The primate cortical LFP exhibits multiple spectral and temporal gradients and widespread task dependence during visual short-term memory. J Neurophysiol.

[110] Mendoza-Halliday D, Major AJ, Lee N, Lichtenfeld MJ, Carlson B, Mitchell B (2024). A ubiquitous spectrolaminar motif of local field potential power across the primate cortex. Nat Neurosci.

[111] Lopes Da Silva FH, Storm Van Leeuwen W (1977). The cortical source of the alpha rhythm. Neurosci Lett.

[112] Lakatos P, Karmos G, Mehta AD, Ulbert I, Schroeder CE (2008). Entrainment of neuronal oscillations as a mechanism of attentional selection. Science.

[113] van Kerkoerle T, Self MW, Dagnino B, Gariel-Mathis MA, Poort J, van der Togt C (2014). Alpha and gamma oscillations characterize feedback and feedforward processing in monkey visual cortex. Proceedings of the National Academy of Sciences of the United States of America.

[114] Dougherty K, Cox MA, Ninomiya T, Leopold DA, Maier A (2017). Ongoing Alpha Activity in V1 Regulates Visually Driven Spiking Responses. Cereb Cortex.

[115] Halgren M, Ulbert I, Bastuji H, Fabó D, Erőss L, Rey M (2019). The generation and propagation of the human alpha rhythm. Proceedings of the National Academy of Sciences.

[116] Markov NT, Vezoli J, Chameau P, Falchier A, Quilodran R, Huissoud C (2014). Anatomy of hierarchy: feedforward and feedback pathways in macaque visual cortex. J Comp Neurol.

[117] Michalareas G, Vezoli J, van Pelt S, Schoffelen JM, Kennedy H, Fries P (2016). Alpha-Beta and Gamma Rhythms Subserve Feedback and Feedforward Influences among Human Visual Cortical Areas. Neuron.

[118] Mackey CA, Duecker K, Neymotin S, Dura-Bernal S, Haegens S, Barczak A (2024). Is there a ubiquitous spectrolaminar motif of local field potential power across primate neocortex?. bioRxiv.

[119] Zhang M, Frohlich F (2022). Cell type-specific excitability probed by optogenetic stimulation depends on the phase of the alpha oscillation. Brain Stimul.

[120] Huang WA, Zhou ZC, Stitt IM, Ramasamy NS, Radtke-Schuller S, Frohlich F (2024). Causal oscillations in the visual thalamo-cortical network in sustained attention in ferrets. Curr Biol.

[121] Schreckenberger M, Lange-Asschenfeld C, Lochmann M, Mann K, Siessmeier T, Buchholz HG (2004). The thalamus as the generator and modulator of EEG alpha rhythm: a combined PET/EEG study with lorazepam challenge in humans. NeuroImage.

[122] Lozano-Soldevilla D (2018). On the Physiological Modulation and Potential Mechanisms Underlying Parieto-Occipital Alpha Oscillations. Front Comput Neurosci.

[123] Jensen O, Goel P, Kopell N, Pohja M, Hari R, Ermentrout B (2005). On the human sensorimotor-cortex beta rhythm: Sources and modeling. NeuroImage.

[124] Nutt D, Wilson S, Lingford-Hughes A, Myers J, Papadopoulos A, Muthukumaraswamy S (2015). Differences between magnetoencephalographic (MEG) spectral profiles of drugs acting on GABA at synaptic and extrasynaptic sites: A study in healthy volunteers. Neuropharmacology.

[125] Baker MR, Baker SN (2003). The effect of diazepam on motor cortical oscillations and corticomuscular coherence studied in man. J Physiol.

[126] Hall SD, Stanford IM, Yamawaki N, McAllister CJ, Rönnqvist KC, Woodhall GL (2011). The role of GABAergic modulation in motor function related neuronal network activity. Neuroimage.

[127] Feshchenko VA, Veselis RA, Reinsel RA (2004). Propofol-induced alpha rhythm. Neuropsychobiology.

[128] Vijayan S, Ching S, Purdon PL, Brown EN, Kopell NJ (2013). Thalamocortical mechanisms for the anteriorization of α rhythms during propofol-induced unconsciousness. J Neurosci.

[129] Hindriks R, van Putten MJAM (2012). Meanfield modeling of propofol-induced changes in spontaneous EEG rhythms. NeuroImage.

[130] Silva LR, Amitai Y, Connors BW (1991). Intrinsic Oscillations of Neocortex Generated by Layer 5 Pyramidal Neurons. Science.

[131] Muthukumaraswamy SD, Shaw AD, Jackson LE, Hall J, Moran R, Saxena N (2015). Evidence that Subanesthetic Doses of Ketamine Cause Sustained Disruptions of NMDA and AMPA-Mediated Frontoparietal Connectivity in Humans. J Neurosci.

[132] Muthukumaraswamy SD, Routley B, Droog W, Singh KD, Hamandi K (2016). The effects of AMPA blockade on the spectral profile of human early visual cortex recordings studied with non-invasive MEG. Cortex.

[133] Rivolta D, Heidegger T, Scheller B, Sauer A, Schaum M, Birkner K (2015). Ketamine Dysregulates the Amplitude and Connectivity of High-Frequency Oscillations in Cortical–Subcortical Networks in Humans: Evidence From Resting-State Magnetoencephalography-Recordings. Schizophrenia Bulletin.

[134] Shaw AD, Saxena N, Jackson E, Hall JE, Singh KD, Muthukumaraswamy SD (2015). Ketamine amplifies induced gamma frequency oscillations in the human cerebral cortex. European Neuropsychopharmacology.

[135] de la Salle S, Choueiry J, Shah D, Bowers H, McIntosh J, Ilivitsky V (2016). Effects of Ketamine on Resting-State EEG Activity and Their Relationship to Perceptual/Dissociative Symptoms in Healthy Humans. Front Pharmacol.

[136] Gilbert DG, Dibb WD, Plath LC, Hiyane SG (2000). Effects of nicotine and caffeine, separately and in combination, on EEG topography, mood, heart rate, cortisol, and vigilance. Psychophysiology.

[137] Bauer M, Kluge C, Bach D, Bradbury D, Heinze HJ, Dolan RJ (2012). Cholinergic Enhancement of Visual Attention and Neural Oscillations in the Human Brain. Current Biology.

[138] Muthukumaraswamy SD, Carhart-Harris RL, Moran RJ, Brookes MJ, Williams TM, Errtizoe D (2013). Broadband Cortical Desynchronization Underlies the Human Psychedelic State. J Neurosci.

[139] Kometer M, Pokorny T, Seifritz E, Volleinweider FX (2015). Psilocybin-induced spiritual experiences and insightfulness are associated with synchronization of neuronal oscillations. Psychopharmacology.

[140] Carhart-Harris RL, Muthukumaraswamy S, Roseman L, Kaelen M, Droog W, Murphy K (2016). Neural correlates of the LSD experience revealed by multimodal neuroimaging. Proceedings of the National Academy of Sciences.

[141] Valle M, Maqueda AE, Rabella M, Rodríguez-Pujadas A, Antonijoan RM, Romero S (2016). Inhibition of alpha oscillations through serotonin-2A receptor activation underlies the visual effects of ayahuasca in humans. European Neuropsychopharmacology.

[142] Dubois J, VanRullen R (2011). Visual Trails: Do the Doors of Perception Open Periodically?. PLOS Biology.

[143] Melgari JM, Curcio G, Mastrolilli F, Salomone G, Trotta L, Tombini M (2014). Alpha and beta EEG power reflects L-dopa acute administration in parkinsonian patients. Frontiers in Aging Neuroscience.

[144] Berger H (1931). Über das Elektrenkephalogramm des Menschen. Archiv f Psychiatrie.

[145] Herning RI, Jones RT, Hooker WD, Mendelson J, Blackwell L (1985). Cocaine increases EEG beta: A replication and extension of Hans Berger’s historic experiments. Electroencephalography and Clinical Neurophysiology.

[146] Albrecht MA, Roberts G, Price G, Lee J, Iyyalol R, Martin-Iverson MT (2016). The effects of dexamphetamine on the resting-state electroencephalogram and functional connectivity. Hum Brain Mapp.

[147] Lopes da Silva FH, Hoeks A, Smits H, Zetterberg LH (1974). Model of brain rhythmic activity. The alpha-rhythm of the thalamus Kybernetik.

[148] VanRullen R, Macdonald JSP (2012). Perceptual echoes at 10 Hz in the human brain. Curr Biol.

[149] Jones SR, Pritchett DL, Sikora MA, Stufflebeam SM, Hämäläinen M, Moore CI (2009). Quantitative Analysis and Biophysically Realistic Neural Modeling of the MEG Mu Rhythm: Rhythmogenesis and Modulation of Sensory-Evoked Responses. Journal of Neurophysiology.

[150] Traub RD, Hawkins K, Adams NE, Hall SP, Simon A, Whittington MA (2020). Layer 4 pyramidal neuron dendritic bursting underlies a post-stimulus visual cortical alpha rhythm. Commun Biol.

[151] Vijayan S, Kopell NJ (2012). Thalamic model of awake alpha oscillations and implications for stimulus processing. Proc Natl Acad Sci U S A.

[152] Naruse Y, Matani A, Miyawaki Y, Okada M (2010). Influence of coherence between multiple cortical columns on alpha rhythm: a computational modeling study. Hum Brain Mapp.

[153] Robinson PA, Loxley PN, O’Connor SC, Rennie CJ (2001). Modal analysis of corticothalamic dynamics, electroencephalographic spectra, and evoked potentials. Phys Rev E Stat Nonlin Soft Matter Phys.

[154] Pfurtscheller G, Neuper C (1994). Event-related synchronization of mu rhythm in the EEG over the cortical hand area in man. Neurosci Lett.

[155] Becker R, Knock S, Ritter P, Jirsa V (2015). Relating Alpha Power and Phase to Population Firing and Hemodynamic Activity Using a Thalamo-cortical Neural Mass Model. PLoS Comput Biol.

[156] Ritter P, Schirner M, McIntosh AR, Jirsa VK (2013). The Virtual Brain Integrates Computational Modeling and Multimodal Neuroimaging. Brain Connectivity.

[157] Bhattacharya BS, Coyle D, Maguire LP (2011). Alpha and theta rhythm abnormality in Alzheimer’s Disease: a study using a computational model. Adv Exp Med Biol.

[158] Palva JM, Monto S, Kulashekhar S, Palva S (2010). Neuronal synchrony reveals working memory networks and predicts individual memory capacity. Proc Natl Acad Sci U S A.

[159] Palva S, Palva JM (2011). Functional roles of alpha-band phase synchronization in local and large-scale cortical networks. Front Psychol.

[160] Siegel M, Donner TH, Oostenveld R, Fries P, Engel AK (2008). Neuronal synchronization along the dorsal visual pathway reflects the focus of spatial attention. Neuron.

[161] Mathewson KE, Beck DM, Ro T, Maclin EL, Low KA, Fabiani M (2014). Dynamics of alpha control: preparatory suppression of posterior alpha oscillations by frontal modulators revealed with combined EEG and event-related optical signal. Journal of cognitive neuroscience.

[162] Marshall TR, Esterer S, Herring JD, Bergmann TO, Jensen O (2015). On the relationship between cortical excitability and visual oscillatory responses - A concurrent tDCS-MEG study. NeuroImage.

[163] Marshall TR, O’Shea J, Jensen O, Bergmann TO (2015). Frontal eye fields control attentional modulation of alpha and gamma oscillations in contralateral occipitoparietal cortex. J Neurosci.

[164] Wang C, Rajagovindan R, Han SM, Ding M (2016). Top-Down Control of Visual Alpha Oscillations: Sources of Control Signals and Their Mechanisms of Action. Front Hum Neurosci.

[165] Popov T, Kastner S, Jensen O (2017). FEF-Controlled Alpha Delay Activity Precedes Stimulus-Induced Gamma-Band Activity in Visual Cortex. J Neurosci.

[166] Marshall TR, Bergmann TO, Jensen O (2015). Frontoparietal Structural Connectivity Mediates the Top-Down Control of Neuronal Synchronization Associated with Selective Attention. PLoS Biol.

[167] Capotosto P, Babiloni C, Romani GL, Corbetta M (2009). Frontoparietal cortex controls spatial attention through modulation of anticipatory alpha rhythms. The Journal of neuroscience : the official journal of the Society for Neuroscience.

[168] Bonnefond M, Jensen O (2024). Resisting Distraction: On the Role of Alpha Oscillations in Gain Modulation and Resource Allocation. OSF.

[169] Jensen O (2024). Distractor inhibition by alpha oscillations is controlled by an in direct mechanism governed by goal-relevant information. Commun Psychol.

[170] Sadaghiani S, Kleinschmidt A (2016). Brain Networks and alpha-Oscillations: Structural and Functional Foundations of Cognitive Control. Trends Cogn Sci.

[171] Solis-Vivanco R, Jensen O, Bonnefond M (2018). Top-Down Control of Alpha Phase Adjustment in Anticipation of Temporally Predictable Visual Stimuli. J Cogn Neurosci.

[172] Gaillard C, Ben Hadj Hassen S, Di Bello F, Bihan-Poudec Y, VanRullen R, Ben Hamed S (2020). Prefrontal attentional saccades explore space rhythmically. Nat Commun.

[173] Albe-Fessard D, Arfel G, Guiot G, Derome P, Hertzog E, Vourc’h G (1966). Electrophysiological studies of some deep cerebral structures in man. J Neurol Sci.

[174] Albe-Fessard D (1973). Electrophysiological methods for the identification of thalamic nuclei. Z Neurol.

[175] Arcaro MJ, Pinsk MA, Chen J, Kastner S (2018). Organizing principles of pulvino-cortical functional coupling in humans. Nat Commun.

[176] Zhou H, Schafer RJ, Desimone R (2016). Pulvinar-Cortex Interactions in Vision and Attention. Neuron.

[177] Fiebelkorn IC, Pinsk MA, Kastner S (2019). The mediodorsal pulvinar coordinates the macaque fronto-parietal network during rhythmic spatial attention. Nat Commun.

[178] Jaramillo J, Mejias JF, Wang XJ (2019). Engagement of Pulvino-cortical Feedforward and Feedback Pathways in Cognitive Computations. Neuron.

[179] Cortes N, Abbas Farishta R, Ladret HJ, Casanova C (2021). Corticothalamic Projections Gate Alpha Rhythms in the Pulvinar. Front Cell Neurosci.

[180] Osipova D, Hermes D, Jensen O (2008). Gamma power is phase-locked to posterior alpha activity. PLoS One.

[181] Olsen SR, Bortone DS, Adesnik H, Scanziani M (2012). Gain control by layer six in cortical circuits of vision. Nature.

[182] Bahramisharif A, van Gerven MA, Aarnoutse EJ, Mercier MR, Schwartz TH, Foxe JJ (2013). Propagating neocortical gamma bursts are coordinated by traveling alpha waves. J Neurosci.

[183] Park H, Lee DS, Kang E, Kang H, Hahm J, Kim JS (2016). Formation of visual memories controlled by gamma power phase-locked to alpha oscillations. Sci Rep.

[184] Bonnefond M, Jensen O (2015). Gamma activity coupled to alpha phase as a mechanism for top-down controlled gating. PLoS One.

[185] Yang X, Fiebelkorn IC, Jensen O, Knight RT, Kastner S (2024). Differential neural mechanisms underlie cortical gating of visual spatial attention mediated by alpha-band oscillations. Proc Natl Acad Sci U S A.

[186] Seymour RA, Rippon G, Gooding-Williams G, Schoffelen JM, Kessler K (2019). Dysregulated oscillatory connectivity in the visual system in autism spectrum disorder. Brain.

[187] Aru J, Aru J, Priesemann V, Wibral M, Lana L, Pipa G (2015). Untangling cross-frequency coupling in neuroscience. Curr Opin Neurobiol.

[188] Jensen O, Spaak E, Park H (2016). Discriminating Valid from Spurious Indices of Phase-Amplitude Coupling. eNeuro.

[189] Seymour RA, Rippon G, Kessler K (2017). The Detection of Phase Amplitude Coupling during Sensory Processing. Front Neurosci.

[190] Haegens S, Nácher V, Hernández A, Luna R, Jensen O, Romo R (2011). Beta oscillations in the monkey sensorimotor network reflect somatosensory decision making. Proc Natl Acad Sci U S A.

[191] Scheeringa R, Mazaheri A, Bojak I, Norris DG, Kleinschmidt A (2011). Modulation of visually evoked cortical FMRI responses by phase of ongoing occipital alpha oscillations. J Neurosci.

[192] Bauer M, Stenner MP, Friston KJ, Dolan RJ (2014). Attentional Modulation of Alpha/Beta and Gamma Oscillations Reflect Functionally Distinct Processes. J Neurosci.

[193] van Ede F, Szebényi S, Maris E (2014). Attentional modulations of somatosensory alpha, beta and gamma oscillations dissociate between anticipation and stimulus processing. Neuroimage.

[194] Palva JM, Palva S, Kaila K (2005). Phase synchrony among neuronal oscillations in the human cortex. J Neurosci.

[195] Palva S, Palva JM (2007). New vistas for alpha-frequency band oscillations. Trends Neurosci.

[196] Sherman MA, Lee S, Law R, Haegens S, Thorn CA, Hämäläinen MS (2016). Neural mechanisms of transient neocortical beta rhythms: Converging evidence from humans, computational modeling, monkeys, and mice. Proc Natl Acad Sci U S A.

[197] Little S, Bonaiuto J, Barnes G, Bestmann S (2019). Human motor cortical beta bursts relate to movement planning and response errors. PLoS Biol.

[198] Bonaiuto JJ, Little S, Neymotin SA, Jones SR, Barnes GR, Bestmann S (2021). Laminar dynamics of high amplitude beta bursts in human motor cortex. Neuroimage.

[199] Enz N, Ruddy KL, Rueda-Delgado LM, Whelan R (2021). Volume of β-Bursts, But Not Their Rate, Predicts Successful Response Inhibition. J Neurosci.

[200] Hummel F, Andres F, Altenmüller E, Dichgans J, Gerloff C (2002). Inhibitory control of acquired motor programmes in the human brain. Brain.

[201] Muthukumaraswamy SD, Myers JFM, Wilson SJ, Nutt DJ, Lingford-Hughes A, Singh KD (2013). The effects of elevated endogenous GABA levels on movement-related network oscillations. Neuroimage.

[202] Fernandez LMJ, Lüthi A (2020). Sleep Spindles: Mechanisms and Functions. Physiol Rev.

[203] Steriade M (1999). Coherent oscillations and short-term plasticity in corticothalamic networks. Trends Neurosci.

[204] Ergenoglu T, Demiralp T, Bayraktaroglu Z, Ergen M, Beydagi H, Uresin Y (2004). Alpha rhythm of the EEG modulates visual detection performance in humans. Brain Res Cogn Brain Res.

[205] Hanslmayr S, Aslan A, Staudigl T, Klimesch W, Herrmann CS, Bäuml KH (2007). Prestimulus oscillations predict visual perception performance between and within subjects. Neuroimage.

[206] van Dijk H, Schoffelen JM, Oostenveld R, Jensen O (2008). Prestimulus oscillatory activity in the alpha band predicts visual discrimination ability. J Neurosci.

[207] Mathewson KE, Gratton G, Fabiani M, Beck DM, Ro T (2009). To see or not to see: prestimulus alpha phase predicts visual awareness. J Neurosci.

[208] Iemi L, Chaumon M, Crouzet SM, Busch NA (2017). Spontaneous Neural Oscillations Bias Perception by Modulating Baseline Excitability. J Neurosci.

[209] Zazio A, Ruhnau P, Weisz N, Wutz A (2022). Pre-stimulus alpha-band power and phase fluctuations originate from different neural sources and exert distinct impact on stimulus-evoked responses. European Journal of Neuroscience.

[210] Samaha J, Iemi L, Haegens S, Busch NA (2020). Spontaneous Brain Oscillations and Perceptual Decision-Making. Trends in Cognitive Sciences.

[211] Linkenkaer-Hansen K, Nikulin VV, Palva S, Ilmoniemi RJ, Palva JM (2004). Prestimulus oscillations enhance psychophysical performance in humans. J Neurosci.

[212] Iemi L, Busch NA, Laudini A, Haegens S, Samaha J, Villringer A (2019). Multiple mechanisms link prestimulus neural oscillations to sensory responses. Elife.

[213] Goldman RI, Stern JM, Engel J, Cohen MS (2002). Simultaneous EEG and fMRI of the alpha rhythm. Neuroreport.

[214] de Munck JC, Gonçalves SI, Mammoliti R, Heethaar RM, Lopes da Silva FH (2009). Interactions between different EEG frequency bands and their effect on alpha–fMRI correlations. NeuroImage.

[215] Liu Y, Bengson J, Huang H, Mangun GR, Ding M (2016). Top-down Modulation of Neural Activity in Anticipatory Visual Attention: Control Mechanisms Revealed by Simultaneous EEG-fMRI. Cereb Cortex.

[216] Mullinger KJ, Cherukara MT, Buxton RB, Francis ST, Mayhew SD (2017). Post-stimulus fMRI and EEG responses: Evidence for a neuronal origin hypothesised to be inhibitory. Neuroimage.

[217] Dugué L, Marque P, VanRullen R (2011). The phase of ongoing oscillations mediates the causal relation between brain excitation and visual perception. J Neurosci.

[218] Busch NA, Dubois J, VanRullen R (2009). The phase of ongoing EEG oscillations predicts visual perception. J Neurosci.

[219] Dou W, Morrow A, Iemi L, Samaha J (2022). Pre-stimulus alpha-band phase gates early visual cortex responses. Neuroimage.

[220] Varela FJ, Toro A, John ER, Schwartz EL (1981). Perceptual framing and cortical alpha rhythm. Neuropsychologia.

[221] Samaha J, Postle BR (2015). The Speed of Alpha-Band Oscillations Predicts the Temporal Resolution of Visual Perception. Curr Biol.

[222] Sharp P, Gutteling T, Melcher D, Hickey C (2022). Spatial Attention Tunes Temporal Processing in Early Visual Cortex by Speeding and Slowing Alpha Oscillations. J Neurosci.

[223] Buergers S, Noppeney U (2022). The role of alpha oscillations in temporal binding within and across the senses. Nat Hum Behav.

[224] Samaha J, Romei V (2023). Alpha-Band Frequency and Temporal Windows in Perception: A Review and Living Meta-analysis of 27 Experiments (and Counting). J Cogn Neurosci.

[225] Schoffelen JM, Pesci UG, Noppeney U (2024). Alpha Oscillations and Temporal Binding Windows in Perception-A Critical Review and Best Practice Guidelines. J Cogn Neurosci.

[226] Worden MS, Foxe JJ, Wang N, Simpson GV (2000). Anticipatory biasing of visuospatial attention indexed by retinotopically specific alpha-band electroencephalography increases over occipital cortex. J Neurosci.

[227] Gould IC, Rushworth MF, Nobre AC (2011). Indexing the graded allocation of visuospatial attention using anticipatory alpha oscillations. J Neurophysiol.

[228] Händel BF, Haarmeier T, Jensen O (2011). Alpha oscillations correlate with the successful inhibition of unattended stimuli. J Cogn Neurosci.

[229] Noonan MP, Adamian N, Pike A, Printzlau F, Crittenden BM, Stokes MG (2016). Distinct Mechanisms for Distractor Suppression and Target Facilitation. J Neurosci.

[230] Zhigalov A, Herring JD, Herpers J, Bergmann TO, Jensen O (2019). Probing cortical excitability using rapid frequency tagging. Neuroimage.

[231] Noonan MP, Crittenden BM, Jensen O, Stokes MG (2018). Selective inhibition of distracting input. Behav Brain Res.

[232] Foster JJ, Awh E (2019). The role of alpha oscillations in spatial attention: limited evidence for a suppression account. Curr Opin Psychol.

[233] van Moorselaar D, Daneshtalab N, Slagter HA (2021). Neural mechanisms underlying distractor inhibition on the basis of feature and/or spatial expectations. Cortex.

[234] Wang B, van Driel J, Ort E, Theeuwes J (2019). Anticipatory Distractor Suppression Elicited by Statistical Regularities in Visual Search. Journal of Cognitive Neuroscience.

[235] van Zoest W, Huber-Huber C, Weaver MD, Hickey C (2021). Strategic Distractor Suppression Improves Selective Control in Human Vision. J Neurosci.

[236] Gutteling TP, Sillekens L, Lavie N, Jensen O (2022). Alpha oscillations reflect suppression of distractors with increased perceptual load. Prog Neurobiol.

[237] Lavie N (2005). Distracted and confused?: selective attention under load. Trends Cogn Sci.

[238] Fiebelkorn IC, Kastner S (2019). A Rhythmic Theory of Attention. Trends Cogn Sci.

[239] Landau AN, Fries P (2012). Attention samples stimuli rhythmically. Curr Biol.

[240] Fiebelkorn IC, Saalmann YB, Kastner S (2013). Rhythmic sampling within and between objects despite sustained attention at a cued location. Curr Biol.

[241] Helfrich RF, Fiebelkorn IC, Szczepanski SM, Lin JJ, Parvizi J, Knight RT (2018). Neural Mechanisms of Sustained Attention Are Rhythmic. Neuron.

[242] Brookshire G (2022). Putative rhythms in attentional switching can be explained by aperiodic temporal structure. Nat Hum Behav.

[243] Fiebelkorn IC, Pinsk MA, Kastner S (2018). A Dynamic Interplay within the Frontoparietal Network Underlies Rhythmic Spatial Attention. Neuron.

[244] Salmelin R, Hari R (1994). Characterization of spontaneous MEG rhythms in healthy adults. Electroencephalogr Clin Neurophysiol.

[245] Haegens S, Nácher V, Luna R, Romo R, Jensen O (2011). α-Oscillations in the monkey sensorimotor network influence discrimination performance by rhythmical inhibition of neuronal spiking. Proceedings of the National Academy of Sciences.

[246] Dockstader C, Cheyne D, Tannock R (2010). Cortical dynamics of selective attention to somatosensory events. Neuroimage.

[247] Jones SR, Kerr CE, Wan Q, Pritchett DL, Hämäläinen M, Moore CI (2010). Cued spatial attention drives functionally relevant modulation of the mu rhythm in primary somatosensory cortex. J Neurosci.

[248] Haegens S, Luther L, Jensen O (2011). Somatosensory Anticipatory Alpha Activity Increases to Suppress Distracting Input. Journal of Cognitive Neuroscience.

[249] Haegens S, Luther L, Jensen O (2012). Somatosensory anticipatory alpha activity increases to suppress distracting input. J Cogn Neurosci.

[250] Whitmarsh S, Barendregt H, Schoffelen JM, Jensen O (2014). Metacognitive awareness of covert somatosensory attention corresponds to contralateral alpha power. Neuroimage.

[251] Wiesman AI, Wilson TW (2020). Attention modulates the gating of primary somatosensory oscillations. Neuroimage.

[252] Weisz N, Hartmann T, Müller N, Lorenz I, Obleser J (2011). Alpha rhythms in audition: cognitive and clinical perspectives. Front Psychol.

[253] Frey JN, Mainy N, Lachaux JP, Müller N, Bertrand O, Weisz N (2014). Selective modulation of auditory cortical alpha activity in an audiovisual spatial attention task. J Neurosci.

[254] Wöstmann M, Alavash M, Obleser J (2019). Alpha Oscillations in the Human Brain Implement Distractor Suppression Independent of Target Selection. J Neurosci.

[255] Kerlin JR, Shahin AJ, Miller LM (2010). Attentional gain control of ongoing cortical speech representations in a ‘cocktail party’. J Neurosci.

[256] Popov T, Gips B, Weisz N, Jensen O (2023). Brain areas associated with visual spatial attention display topographic organization during auditory spatial attention. Cereb Cortex.

[257] Gomez-Ramirez M, Kelly SP, Molholm S, Sehatpour P, Schwartz TH, Foxe JJ (2011). Oscillatory sensory selection mechanisms during intersensory attention to rhythmic auditory and visual inputs: a human electrocorticographic investigation. J Neurosci.

[258] Banerjee S, Snyder AC, Molholm S, Foxe JJ (2011). Oscillatory alpha-band mechanisms and the deployment of spatial attention to anticipated auditory and visual target locations: supramodal or sensory-specific control mechanisms?. J Neurosci.

[259] Mazaheri A, van Schouwenburg MR, Dimitrijevic A, Denys D, Cools R, Jensen O (2014). Region-specific modulations in oscillatory alpha activity serve to facilitate processing in the visual and auditory modalities. Neuroimage.

[260] Haegens S, Osipova D, Oostenveld R, Jensen O (2010). Somatosensory working memory performance in humans depends on both engagement and disengagement of regions in a distributed network. Hum Brain Mapp.

[261] Jokisch D, Jensen O (2007). Modulation of gamma and alpha activity during a working memory task engaging the dorsal or ventral stream. J Neurosci.

[262] Snyder AC, Foxe JJ (2010). Anticipatory attentional suppression of visual features indexed by oscillatory alpha-band power increases: a high-density electrical mapping study. J Neurosci.

[263] Tuladhar AM, ter Huurne N, Schoffelen JM, Maris E, Oostenveld R, Jensen O (2007). Parieto-occipital sources account for the increase in alpha activity with working memory load. Hum Brain Mapp.

[264] Magosso E, Borra D (2024). The strength of anticipated distractors shapes EEG alpha and theta oscillations in a Working Memory task. NeuroImage.

[265] Tu CA, Parviainen T, Hämäläinen JA, Hsu YF (2025). Alpha oscillations protect auditory working memory against distractors in the encoding phase. Neuropsychologia.

[266] Myers NE, Walther L, Wallis G, Stokes MG, Nobre AC (2015). Temporal dynamics of attention during encoding versus maintenance of working memory: complementary views from event-related potentials and alpha-band oscillations. J Cogn Neurosci.

[267] Mok RM, Myers NE, Wallis G, Nobre AC (2016). Behavioral and Neural Markers of Flexible Attention over Working Memory in Aging. Cereb Cortex.

[268] Bahramisharif A, Jensen O, Jacobs J, Lisman J (2018). Serial representation of items during working memory maintenance at letter-selective cortical sites. PLOS Biology.

[269] Park H, Lee DS, Kang E, Kang H, Hahm J, Kim JS (2014). Blocking of irrelevant memories by posterior alpha activity boosts memory encoding. Hum Brain Mapp.

[270] Meeuwissen EB, Takashima A, Fernández G, Jensen O (2011). Increase in posterior alpha activity during rehearsal predicts successful long-term memory formation of word sequences. Hum Brain Mapp.

[271] Stokes MG, Atherton K, Patai EZ, Nobre AC (2012). Long-term memory prepares neural activity for perception. Proc Natl Acad Sci U S A.

[272] Klimesch W, Doppelmayr M, Pachinger T, Ripper B (1997). Brain oscillations and human memory: EEG correlates in the upper alpha and theta band. Neuroscience Letters.

[273] Hanslmayr S, Staresina BP, Bowman H (2016). Oscillations and Episodic Memory: Addressing the Synchronization/Desynchronization Conundrum. Trends in Neurosciences.

[274] Wang L, Jensen O, van den Brink D, Weder N, Schoffelen JM, Magyari L (2012). Beta oscillations relate to the N400m during language comprehension. Hum Brain Mapp.

[275] Rommers J, Dickson DS, Norton JJS, Wlotko EW, Federmeier KD (2017). Alpha and theta band dynamics related to sentential constraint and word expectancy. Language, Cognition and Neuroscience.

[276] Wang L, Hagoort P, Jensen O (2018). Language Prediction Is Reflected by Coupling between Frontal Gamma and Posterior Alpha Oscillations. J Cogn Neurosci.

[277] León-Cabrera P, Piai V, Morís J, Rodríguez-Fornells A (2022). Alpha power decreases associated with prediction in written and spoken sentence comprehension. Neuropsychologia.

[278] Terporten R, Huizeling E, Heidlmayr K, Hagoort P, Kösem A (2024). The Interaction of Context Constraints and Predictive Validity during Sentence Reading. J Cogn Neurosci.

[279] Obleser J, Weisz N (2012). Suppressed alpha oscillations predict intelligibility of speech and its acoustic details. Cereb Cortex.

[280] Wöstmann M, Lim SJ, Obleser J (2017). The Human Neural Alpha Response to Speech is a Proxy of Attentional Control. Cereb Cortex.

[281] Başar E, Başar-Eroğlu C, Güntekin B, Yener GG (2013). Brain’s alpha, beta, gamma, delta, and theta oscillations in neuropsychiatric diseases: proposal for biomarker strategies. Suppl Clin Neurophysiol.

[282] Oswal A, Brown P, Litvak V (2013). Synchronized neural oscillations and the pathophysiology of Parkinson’s disease. Curr Opin Neurol.

[283] Ippolito G, Bertaccini R, Tarasi L, Di Gregorio F, Trajkovic J, Battaglia S (2022). The Role of Alpha Oscillations among the Main Neuropsychiatric Disorders in the Adult and Developing Human Brain: Evidence from the Last 10 Years of Research. Biomedicines.

[284] Giustiniani A, Danesin L, Bozzetto B, Macina A, Benavides-Varela S, Burgio F (2023). Functional changes in brain oscillations in dementia: a review. Rev Neurosci.

[285] Mazaheri A, Fassbender C, Coffey-Corina S, Hartanto TA, Schweitzer JB, Mangun GR (2014). Differential oscillatory electroencephalogram between attention-deficit/hyperactivity disorder subtypes and typically developing adolescents. Biol Psychiatry.

[286] Lenartowicz A, Mazaheri A, Jensen O, Loo SK (2018). Aberrant Modulation of Brain Oscillatory Activity and Attentional Impairment in Attention-Deficit/Hyperactivity Disorder. Biol Psychiatry Cogn Neurosci Neuroimaging.

[287] ter Huurne N, Onnink M, Kan C, Franke B, Buitelaar J, Jensen O (2013). Behavioral consequences of aberrant alpha lateralization in attention-deficit/hyperactivity disorder. Biol Psychiatry.

[288] Vollebregt MA, Zumer JM, Ter Huurne N, Buitelaar JK, Jensen O (2016). Posterior alpha oscillations reflect attentional problems in boys with Attention Deficit Hyperactivity Disorder. Clin Neurophysiol.

[289] Lenartowicz A, Delorme A, Walshaw PD, Cho AL, Bilder RM, McGough JJ (2014). Electroencephalography correlates of spatial working memory deficits in attention-deficit/hyperactivity disorder: vigilance, encoding, and maintenance. J Neurosci.

[290] Lenartowicz A, Truong H, Salgari GC, Bilder RM, McGough J, McCracken JT (2019). Alpha modulation during working memory encoding predicts neurocognitive impairment in ADHD. J Child Psychol Psychiatry.

[291] Mazaheri A, Coffey-Corina S, Mangun GR, Bekker EM, Berry AS, Corbett BA (2010). Functional disconnection of frontal cortex and visual cortex in attention-deficit/hyperactivity disorder. Biol Psychiatry.

[292] Michelini G, Salmastyan G, Vera JD, Lenartowicz A (2022). Event-related brain oscillations in attention-deficit/hyperactivity disorder (ADHD): A systematic review and meta-analysis. Int J Psychophysiol.

[293] Leenders MP, Lozano-Soldevilla D, Roberts MJ, Jensen O, De Weerd P (2018). Diminished Alpha Lateralization During Working Memory but Not During Attentional Cueing in Older Adults. Cereb Cortex.

[294] Pinal D, Zurrón M, Díaz F, Sauseng P (2015). Stuck in default mode: inefficient cross-frequency synchronization may lead to age-related short-term memory decline. Neurobiol Aging.

[295] Jafari Z, Kolb BE, Mohajerani MH (2020). Neural oscillations and brain stimulation in Alzheimer’s disease. Prog Neurobiol.

[296] Babiloni C, Arakaki X, Bonanni L, Bujan A, Carrillo MC, Del Percio C (2021). EEG measures for clinical research in major vascular cognitive impairment: recommendations by an expert panel. Neurobiol Aging.

[297] Nunez PL (1974). The brain wave equation: a model for the EEG. Mathematical Biosciences.

[298] Ermentrout GB, Kleinfeld D (2001). Traveling electrical waves in cortex: insights from phase dynamics and speculation on a computational role. Neuron.

[299] Muller L, Chavane F, Reynolds J, Sejnowski TJ (2018). Cortical travelling waves: mechanisms and computational principles. Nat Rev Neurosci.

[300] Zhang H, Watrous AJ, Patel A, Jacobs J (2018). Theta and Alpha Oscillations Are Traveling Waves in the Human Neocortex. Neuron.

[301] Kaneko T, Komatsu M, Yamamori T, Ichinohe N, Okano H (2022). Cortical neural dynamics unveil the rhythm of natural visual behavior in marmosets. Commun Biol.

[302] Pang Z, Alamia A, VanRullen R (2020). Turning the Stimulus On and Off Changes the Direction of α Traveling Waves. eNeuro.

[303] Alamia A, Terral L, D’ambra MR, VanRullen R, Obleser J, Baker CI, Keitel C (2023). Distinct roles of forward and backward alpha-band waves in spatial visual attention. eLife.

[304] Alamia A, VanRullen R (2019). Alpha oscillations and traveling waves: Signatures of predictive coding?. PLOS Biology.

[305] Zhigalov A, Jensen O (2023). Perceptual echoes as travelling waves may arise from two discrete neuronal sources. NeuroImage.

[306] Grabot L, Merholz G, Winawer J, Heeger DJ, Dugué L (2024). Traveling Waves in the Human Visual Cortex: a MEG-EEG Model-Based Approach. bioRxiv.

[307] Patten TM, Rennie CJ, Robinson PA, Gong P (2012). Human cortical traveling waves: dynamical properties and correlations with responses. PLoS One.

[308] Townsend RG, Solomon SS, Chen SC, Pietersen ANJ, Martin PR, Solomon SG (2015). Emergence of complex wave patterns in primate cerebral cortex. J Neurosci.

[309] Deco G, Kringelbach ML (2020). Turbulent-like Dynamics in the Human Brain. Cell Rep.

[310] Sanz Perl Y, Escrichs A, Tagliazucchi E, Kringelbach ML, Deco G (2022). Strength-dependent perturbation of whole-brain model working in different regimes reveals the role of fluctuations in brain dynamics. PLoS Comput Biol.

[311] Drewes J, VanRullen R (2011). This Is the Rhythm of Your Eyes: The Phase of Ongoing Electroencephalogram Oscillations Modulates Saccadic Reaction Time. J Neurosci.

[312] Staudigl T, Hartl E, Noachtar S, Doeller CF, Jensen O (2017). Saccades are phase-locked to alpha oscillations in the occipital and medial temporal lobe during successful memory encoding. PLoS Biol.

[313] Pan Y, Popov T, Frisson S, Jensen O (2023). Saccades are locked to the phase of alpha oscillations during natural reading. PLOS Biology.

[314] Neupane S, Guitton D, Pack CC (2017). Coherent alpha oscillations link current and future receptive fields during saccades. Proceedings of the National Academy of Sciences.

[315] Bellet J, Chen CY, Hafed ZM (2017). Sequential hemifield gating of α- and β-behavioral performance oscillations after microsaccades. J Neurophysiol.

[316] Liu B, Nobre AC, van Ede F (2022). Functional but not obligatory link between microsaccades and neural modulation by covert spatial attention. Nat Commun.

[317] Liu B, Nobre AC, van Ede F (2023). Microsaccades transiently lateralise EEG alpha activity. Prog Neurobiol.

[318] Wang S, van Ede F (2025). Re-focusing visual working memory during expected and unexpected memory tests. Elife.

[319] Popov T, Staudigl T (2023). Cortico-ocular coupling in the service of episodic memory formation. Prog Neurobiol.

[320] Boto E, Holmes N, Leggett J, Roberts G, Shah V, Meyer SS (2018). Moving magnetoencephalography towards real-world applications with a wearable system. Nature.

[321] Knyazeva MG, Barzegaran E, Vildavski VY, Demonet JF (2018). Aging of human alpha rhythm. Neurobiol Aging.

[322] Cesnaite E, Steinfath P, Jamshidi Idaji M, Stephani T, Kumral D, Haufe S (2023). Alterations in rhythmic and non-rhythmic resting-state EEG activity and their link to cognition in older age. NeuroImage.

[323] Ünsal E, Duygun R, Yemeniciler İ, Bingöl E, Ceran Ö, Güntekin B (2024). From Infancy to Childhood: A Comprehensive Review of Event- and Task-Related Brain Oscillations. Brain Sci.

[324] Marshall PJ, Bar-Haim Y, Fox NA (2002). Development of the EEG from 5 months to 4 years of age. Clin Neurophysiol.

[325] Cellier D, Riddle J, Petersen I, Hwang K (2021). The development of theta and alpha neural oscillations from ages 3 to 24 years. Dev Cogn Neurosci.

[326] Tröndle M, Popov T, Dziemian S, Langer N, Dugué L, Shinn-Cunningham BG, Li W, Donoghue T (2022). Decomposing the role of alpha oscillations during brain maturation. eLife.

[327] Caffarra S, Kanopka K, Kruper J, Richie-Halford A, Roy E, Rokem A (2024). Development of the Alpha Rhythm Is Linked to Visual White Matter Pathways and Visual Detection Performance. J Neurosci.

[328] Suldo SM, Olson LA, Evans JR (2002). Quantitative EEG Evidence of Increased Alpha Peak Frequency in Children with Precocious Reading Ability. Journal of Neurotherapy.

[329] Babiloni C, Binetti G, Cassetta E, Dal Forno G, Del Percio C, Ferreri F (2006). Sources of cortical rhythms change as a function of cognitive impairment in pathological aging: a multicenter study. Clin Neurophysiol.

[330] Kumral D, Cesnaite E, Beyer F, Hofmann SM, Hensch T, Sander C (2022). Relationship between regional white matter hyperintensities and alpha oscillations in older adults. Neurobiol Aging.

[331] Hill RM, Boto E, Holmes N, Hartley C, Seedat ZA, Leggett J (2019). A tool for functional brain imaging with lifespan compliance. Nat Commun.

[332] Zahran S, Mahmoudzadeh M, Wallois F, Betrouni N, Derambure P, Le Prado M (2022). Performance Analysis of Optically Pumped 4He Magnetometers vs. Conventional SQUIDs: From Adult to Infant Head Models. Sensors (Basel).

[333] Feys O, De Tiège X (2024). From cryogenic to on-scalp magnetoencephalography for the evaluation of paediatric epilepsy. Developmental Medicine & Child Neurology.

[334] Rhodes N, Sato J, Safar K, Amorim K, Taylor MJ, Brookes MJ (2024). Paediatric magnetoencephalography and its role in neurodevelopmental disorders. Br J Radiol.

[335] Vogel F (1970). The genetic basis of the normal human electroencephalogram (EEG). Humangenetik.

[336] Young JP, Lader MH, Fenton GW (1972). A twin study of the genetic influences on the electroencephalogram. Journal of Medical Genetics.

[337] Lykken DT, Tellegen A, Iacono WG (1982). EEG spectra in twins: Evidence for a neglected mechanism of genetic determination. Psychobiology.

[338] van Beijsterveldt CE, Molenaar PC, de Geus EJ, Boomsma DI (1996). Heritability of human brain functioning as assessed by electroencephalography. Am J Hum Genet.

[339] Posthuma D, Neale MC, Boomsma DI, de Geus EJ (2001). Are smarter brains running faster? Heritability of alpha peak frequency, IQ, and their interrelation. Behav Genet.

[340] van Beijsterveldt CEM, van Baal GCM (2002). Twin and family studies of the human electroencephalogram: a review and a meta-analysis. Biol Psychol.

[341] Smit CM, Wright MJ, Hansell NK, Geffen GM, Martin NG (2006). Genetic variation of individual alpha frequency (IAF) and alpha power in a large adolescent twin sample. Int J Psychophysiol.

